# Thermal Insulation Performance of Aerogel Nano-Porous Materials: Characterization and Test Methods

**DOI:** 10.3390/gels9030220

**Published:** 2023-03-14

**Authors:** Fengfei Lou, Sujun Dong, Keyong Zhu, Xiaona Chen, Yinwei Ma

**Affiliations:** 1 School of Aeronautical Science and Engineering, Beihang University, Beijing 100083, China; lloufengfei@163.com (F.L.); dsj@buaa.edu.cn (S.D.); 2 Research Department of Airframe Technology, Beijing Institute of Aerospace Technology, Beijing 100074, China; chenxiaona228@163.com (X.C.); riskyma@163.com (Y.M.)

**Keywords:** nano-porous insulation material, aerogel, effective thermal conductivity, characterization method, test method

## Abstract

Due to the extremely high porosity and extremely low density of nano-porous thermal insulation materials, the characteristic size of the pores inside the materials and the characteristic size of the solid skeleton structure are on the nanometer scale, which leads to the obvious nanoscale effect of the heat transfer law inside the aerogel materials. Therefore, the nanoscale heat transfer characteristics inside the aerogel materials and the existing mathematical models for calculating the thermal conductivity of various heat transfer modes at the nanoscale need to be summarized in detail. Moreover, in order to verify the accuracy of the thermal conductivity calculation model of aerogel nano-porous materials, correct experimental data are required to modify the model. Because the medium is involved in radiation heat transfer, the existing test methods have a large error, which brings great difficulties to the design of nano-porous materials. In this paper, the heat transfer mechanism, characterization methods, and test methods of thermal conductivity of nano-porous materials are summarized and discussed. The main contents of this review are as follows. The first part introduces the structural characteristics and specific application environment of aerogel. In the second part, the characteristics of nanoscale heat transfer of aerogel insulation materials are analyzed. In the third part, the characterization methods of thermal conductivity of aerogel insulation materials are summarized. In the fourth part, the test methods of thermal conductivity of aerogel insulation materials are summarized. The fifth part gives a brief conclusion and prospect.

## 1. The Structural Characteristics

**Figure 1:**
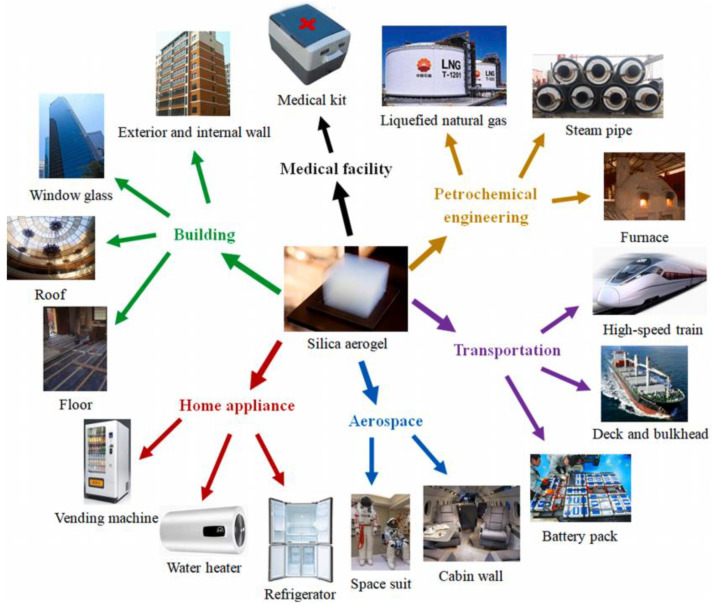
Application range of aerogel [3].

According to the pore size, porous materials are divided into four categories by the International Union of Pure and Applied Chemistry (IUPAC) [1]: Micropore, with the pore size ≤2 nm; Mesopore, with the pore size of 2–50 nm; Macropore, with the pore size of 50–1000 nm; Pore, with the pore size ≥1000 nm. In recent years, mesoporous materials with nanoscale structure have attracted great attention in international physics, chemistry, and materials, and have rapidly developed into one of the interdisciplinary research hotspots. Aerogel is a typical mesoporous material, belonging to solid material with a disordered mesoporous shape that is irregular but interconnected [2]. Aerogel is a kind of mesoporous amorphous material with a very high specific surface area (>800 m^2^/g) and very low density (<100 kg/m^3^), and its porosity can reach more than 80%. Because of the nanoscale pore size of aerogel, the thermal conductivity at normal temperature and pressure is lower than that of air in free space. Aerogel is also known as super insulation material because of its unparalleled thermal insulation performance. As shown in Figure 1 [3], aerogel is widely used in aerospace, energy, chemical industry, construction and other fields.

**Figure 2:**
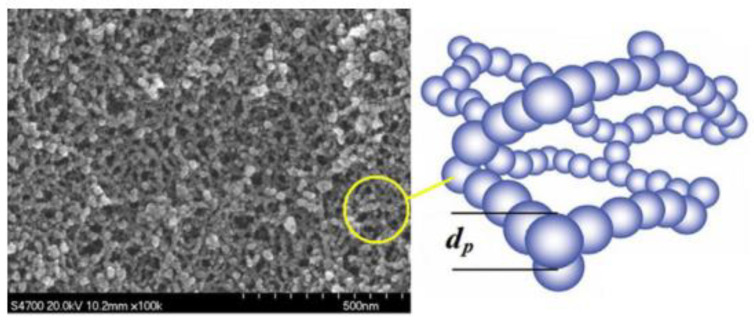
Structure of typical nano-porous insulation materials: SEM image of silica aerogel ($d_{p}$ represents the diameter of the particle).

The extremely low thermal conductivity of aerogel nano-porous insulation materials is caused by their inherent complex nanostructures, as shown in Figure 2 [4]. The nanoparticles of the nano-porous materials randomly gather together to form chain skeletons, which are connected to each other to form a complex three-dimensional network structure. Firstly, the structure generates a large number of nanopores, and the pore size inside aerogel is smaller than or close to the mean free path of gas molecules. Such nanoscale pores will severely restrict the free movement of gas molecules, and the heat transfer between gas molecules will be severely weakened [5,6]. Secondly, when heat is transferred through the solid skeleton, the complex skeleton structure of the nano-porous insulation materials increases the heat transfer path, which makes the aerogel nano-porous insulation materials produce large thermal resistance, and thus the solid thermal conductivity is lower. Finally, it must be mentioned that aerogel has strong permeability to near-infrared radiation with wavelengths of 3–8 μm at high temperature, which leads to the poor shielding ability of aerogel at high temperature, and the thermal conductivity of aerogel increases significantly with the increase in temperature [7,8].

To sum up, the basic structure of aerogel materials consists of nanoscale pores and nanoscale solid skeleton structures, and the nanoscale heat transfer problem is very complicated. Therefore, it is of great significance to study the heat transfer mode inside aerogel insulation materials in detail and reveal the heat transfer mechanism for further reducing the heat transfer performance of nano-porous thermal insulation materials. Moreover, in order to verify the accuracy of the thermal conductivity calculation model of aerogel nano-porous materials, correct experimental data are required to modify the model. Because the medium is involved in radiation heat transfer, the existing test methods have a large error, which brings great difficulties to the design of nano-porous materials. In this paper, the heat transfer mechanism, characterization method, and thermal conductivity measurement method of nano-porous materials are reviewed and discussed, which can provide some help for the subsequent research on the heat transfer calculation model, test methods, and engineering rapid calculation of the equivalent thermal conductivity of aerogel materials.

## 2. Analysis on Heat Transfer Mechanism

**Figure 3:**
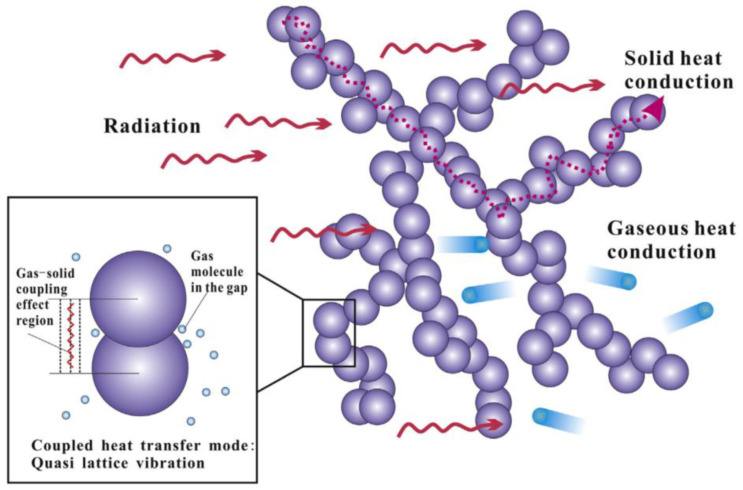
Heat transfer modes of aerogel insulation materials.

**Figure 4:**
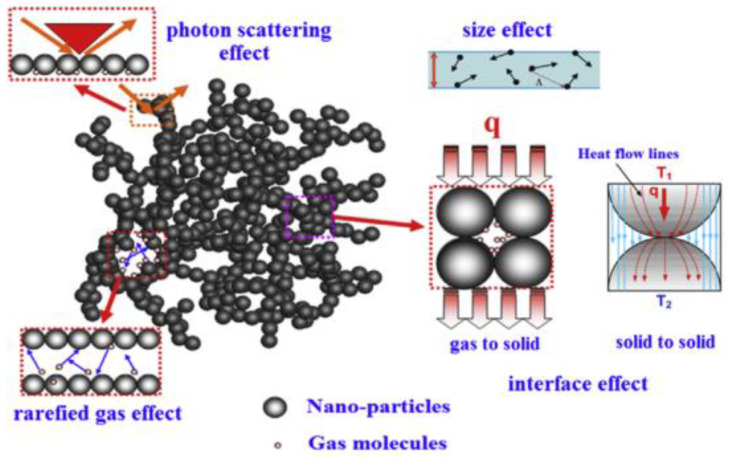
Common heat transfer effects of aerogel insulation materials.

As shown in Figure 3 [9], heat transfer modes inside aerogel materials can be summarized into three modes: (1) Heat transfer between different gas molecules (blue particles); (2) Heat transfer along the solid skeleton structure (red dotted line); (3) Radiant heat transfer (red wavy arrow). In addition, convection is the macroscopic motion of fluid, which causes relative displacement among all parts of fluid, resulting in the mixing of cold and hot fluids, and thus generating heat transfer. It has been reported that for porous materials, when the pore diameter of the material is less than 4 mm, the heat transfer generated by fluid convection can be basically ignored [10,11]. The internal pore size of aerogel materials is basically on the scale of nanoscale, so the convection can be ignored when considering the law of heat transfer inside aerogel, that is, heat conduction and thermal radiation are the two main heat transfer modes of aerogel insulation materials [12]. According to the nanoscale heat transfer theory, many factors significantly affect heat transfer in nano-porous materials. For example, size effect will reduce the heat conduction of aerogel solid skeleton, pore size effect and rarefied gas effect will both reduce the heat transfer of gas molecules, and photon scattering will also affect the radiation heat transfer. Common effects are shown in Figure 4 [13]. The specific analysis of the two heat transfer modes of heat conduction and heat radiation is as follows:

As aerogel nano-porous materials are composed of gas phase component and solid phase component, heat conduction inside aerogel can be subdivided into gas thermal conductivity and solid thermal conductivity [14,15].

**Figure 5:**
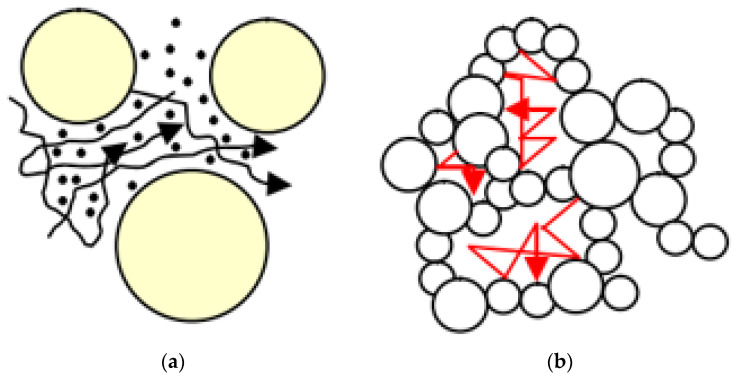
Gas thermal conductivity: (**a**) General porous materials; (**b**) Aerogel nano-porous materials.

Gas thermal conductivity refers to the heat transfer generated by the collision of gas molecules in pores. The gas heat conduction in general porous materials and aerogel nano-porous materials is shown in Figure 5 [16]. The pore size of aerogel is generally 5~100 nm, and the mean pore size is about 20~40 nm [17], while the mean free path of air in standard state is about 69 nm. It can be seen that the pore diameter inside aerogel is smaller than or close to the mean free path of gas molecules, and such nanoscale pores will severely restrict the free movement of molecules, thus greatly reducing the gas thermal conductivity [18].

**Figure 6:**
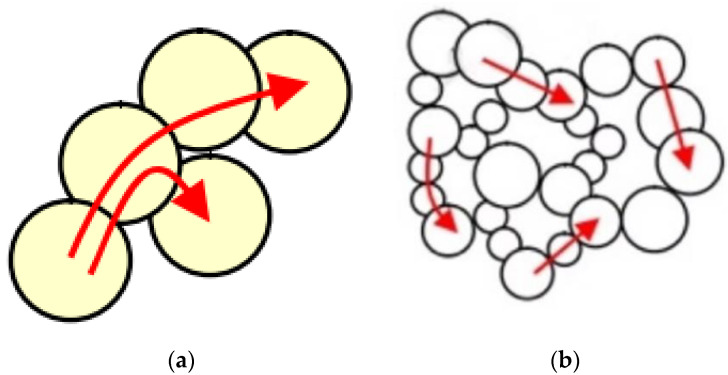
Solid thermal conductivity: (**a**) General porous materials; (**b**) Aerogel nano-porous materials.

The solid thermal conductivity mainly depends on the lattice vibration of solid molecules at the equilibrium position. Taking silica aerogel as an example, primary particle diameter generally ranges from 2 to 5 nm [19], while for amorphous silica materials, the mean free path of the phonon is about 0.58 nm [4,20]. It can be seen that, for aerogel materials, the characteristic scale of the skeleton is also close to the mean free path of solid phonons, so the solid thermal conductivity is significantly reduced under the effect of nanoscale [21]. Solid thermal conductivity in general porous materials and aerogel nano-porous materials is shown in Figure 6. Generally, porous materials have a large particle contact area and a short path, while aerogel nano-porous materials have a small particle contact area, and heat transfer through a complex path, thus extending the heat transfer path, which is the second reason for the lower solid thermal conductivity of aerogel.

For aerogel nano-porous materials, the radiation heat imported from the outside or emitted by the solid skeleton in the nano-porous material will be absorbed and scattered by the solid phase material when it passes through the pores or skeleton in the nano-porous material [22,23]. It must be mentioned that aerogel has strong permeability to near-infrared radiation with wavelengths of 3–8 μm at high temperature, which leads to the poor shielding ability of aerogel at high temperature, and the thermal conductivity of aerogel increases significantly with the increase in temperature [24].

**Figure 7:**
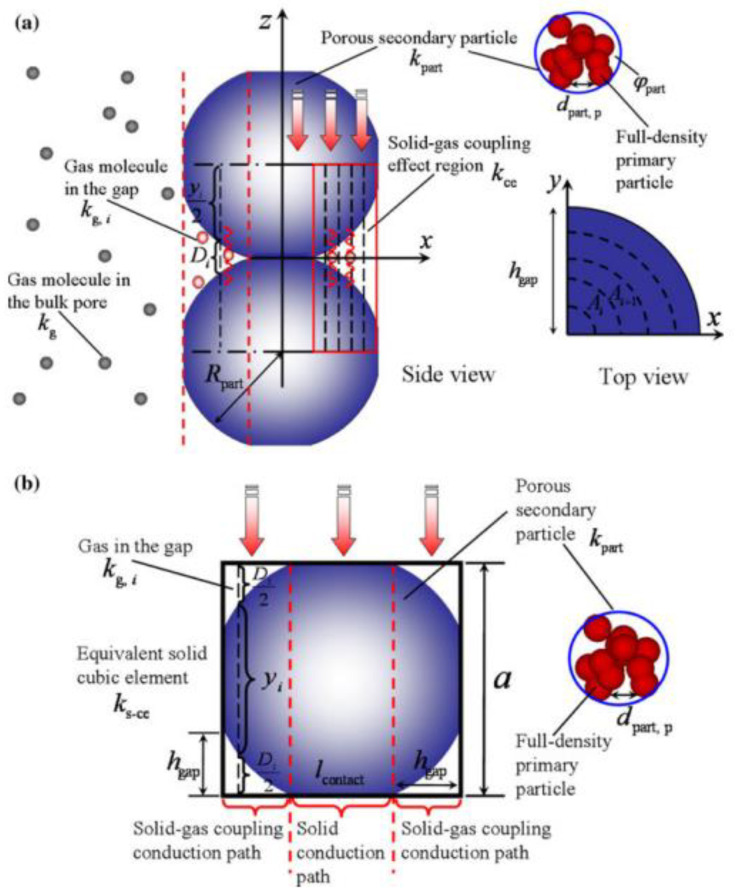
Gap between gas-solid coupling heat transfer and adjacent porous secondary nanoparticles (*a*, *D*, *Ai* and *d* represent equivalent solid cubic element length, pore diameter, ring area of the *i*th hollow cylinder and particle size, respectively.): (**a**) Cylindrical element in gas-solid coupling effect zone; (**b**) Equivalent solid cubic element.

It should be mentioned here that in addition to the three basic heat transfer modes mentioned above, namely gas phase heat transfer, solid phase heat transfer and radiation heat transfer, gas-solid coupled heat transfer is also considered to be an important heat transfer mode. Due to the nanoscale pores and solid skeleton of aerogel, a large number of gas molecules are gathered in the contact surface of solid particles, forming the gas-solid coupling heat transfer effect and enhancing the effective heat transfer of materials, as shown in Figure 7 [25]. In order to show the characteristics of various heat transfer models of aerogel more clearly, the heat-transfer characteristics of various models are summarized in Table 1.

In conclusion, because aerogel nano-porous insulation materials have extremely high porosity and extremely low density, the characteristic size of the pore and solid skeleton structure inside the material is on the nanometer scale, which is close to or smaller than the mean free path of the corresponding energy carrier. Therefore, the heat-transfer law inside aerogel has obvious nanoscale effect. At the same time, the influence of heat transfer on aerogel materials will also be affected by gas-solid coupled heat transfer as shown in Figure 7. Therefore, different heat transfer modes of aerogel insulation materials at the nanoscale should be studied, and the characterization and test methods of thermal conductivity of nano-porous insulation materials should be summarized.

## 3. Characterization Methods of Thermal Insulation Performance

For the existing calculation model of thermal conductivity of aerogel materials, this chapter will be introduced in two parts. The first part is introduced according to the four basic heat transfer modes contained in the material: gas phase heat transfer, solid phase heat transfer, gas-solid coupling heat transfer and radiation heat transfer. The second part is a summary of the overall thermal conductivity of aerogel nano-porous materials. The thermal conductivity corresponding to the overall thermal insulation performance of materials refers to the effective thermal conductivity.

### 3.1. Calculation Models of Gas, Solid, Gas-Solid Coupling, and Radiation

#### 3.1.1. Calculation Model of Gas Thermal Conductivity

There are roughly three methods for calculating the gas thermal conductivity of aerogels. First, based on the experimental data, the gas thermal conductivity is fitted to the empirical formula of related parameters, such as the empirical formula related to the aerogel density. The second method employs the Lattice Boltzmann Method (LBM), Direct Simulation Monte Carlo (DSMC) or Molecular Dynamics (MD), and other numerical simulation methods to obtain the law of gas phase heat transfer of aerogel nano-porous materials. Thirdly, based on the theory of molecular motion, the gas thermal conductivity of nano-porous materials is derived theoretically [26,27].

##### Empirical Correlation Formula

Since the pore size varies with the density of aerogel, the gas thermal conductivity of aerogel is fitted into the empirical correlation formula of material density based on the measurement results [28], as shown in Equation (1):

(1) $$\lambda_{g}\propto \rho_{a}^{-0.6}$$

where $\lambda_{g}$ is the gas thermal conductivity and $\rho_{a}$ is the apparent density of the aerogel.

The disadvantage of the empirical correlation formula shown in Equation (1) is that it lacks strict theoretical basis. The calculation equation of gas thermal conductivity is only affected by macroscopic density and cannot reflect the influence of microscopic solid skeleton structure and pore size on gas thermal conductivity, nor can it reveal the change law of gas thermal conductivity with pressure and temperature.

##### Numerical Simulation Method

As for the numerical simulation method, because there are nanoscale pores in aerogel insulation materials, the numerical research method of gas phase heat transfer at the micro- and nanoscale is used to calculate gas thermal conductivity, such as LBM, DSMC, MD, etc. [29]. LBM is directly used to solve the Boltzmann transport equation, which is a tool used to analyze the micro-scale energy transport phenomenon [30,31]. The Boltzmann transport equation is shown in Equation (2).

(2) $$\frac{\partial f}{\partial t}+v\frac{\partial f}{\partial r}+F\frac{\partial f}{\partial p}={\left(\frac{\partial f}{\partial t}\right)}_{\mathrm{scat}\;}$$

where *f* is the statistical distribution function of particles, which is related to time *t*, position vector **r** and momentum **p**, **F** is the force acting on the particles, and **v** is the particle velocity.

##### Theoretical Derivation Method

The Kaganer model [32] is widely used to calculate the gas thermal conductivity in nanoscale pores. Based on the assumption of parallel plates, the pore size is equivalent to the distance between parallel plates, thus revealing the influence of pore size on the gas thermal conductivity. The specific formula is as follows:

(3) $$\lambda_{g}=\frac{\lambda_{0}}{1+2\beta Kn}$$

(4) $$K_{n}=l_{m}/D$$

(5) $$l_{m}=K_{B}T/\left(\sqrt{2}\pi d_{g}^{2}p\right)$$

where $\lambda_{0}$ is the gas thermal conductivity in free space, $K_{n}$ is the Knudsen number, which is the ratio of the mean free path $l_{m}$ of gas molecules to the characteristic dimension *D* of pores, $d_{g}$ is the effective diameter of gas molecules, $K_{B}$ is the Boltzmann constant, p and T are pressure and temperature, respectively.

In the Kaganer model, the mean free path of gas molecules is considered to describe the effect of pore size on gas thermal conductivity, which can reveal the effect of temperature and pressure on gas thermal conductivity. Therefore, the Kaganer model has been widely used.

However, in the Kaganer model, the influence of pore size in the parameter *D* is not considered. For aerogel nano-porous insulation materials, the pore size distribution inside the aerogel has a significant impact on the gas thermal conductivity. Because the pore size distribution in aerogel materials is not uniform, if the average pore size is directly used to calculate the gas phase thermal conductivity, there will be a large deviation. Therefore, Reichenauer et al. [33] and Bi et al. [34] modified the pore size to a certain extent based on the Kaganer model.

Pore size correction model 1: In Kaganer model, the mean pore size is used to describe the pore distribution inside the material, which is suitable to describe the nano-porous materials with a concentrated pore size distribution. In order to describe nano-porous materials with multiple characteristic pore sizes, Reichenauer et al. [33] in 2007 proposed a calculation model of gas thermal conductivity of nano-porous materials based on double pore size distribution:

(6) $$\lambda_{g}=\frac{\lambda_{0}^{}\varphi_{1}}{1+2\beta l_{m}/D_{1}}+\frac{\lambda_{0}^{}\varphi_{2}}{1+2\beta l_{m}/D_{2}}$$

where *D*_1_ and *D*_2_ are two characteristic pore sizes used to describe the pore size distribution of nano-porous materials, respectively, $\varphi_{1}$ and $\varphi_{2}$ are the proportions of the two characteristic pore sizes, respectively.

The authors considered more detailed pore size distribution in the material, so Equation (6) is modified. Reichenauer et al. [33] assumed that the pore size distribution in nano-porous materials is the Gauss distribution, and then weighted summation of the gas thermal conductivity within each pore size is performed to obtain the gas thermal conductivity, as shown in the following formulas, which are called the Gaussian model.

(7) $$\lambda_{g}=\sum_{i=1}^{n}\Phi_{i}K\left(D_{i}\right)$$

(8) $$\begin{array}{l} \Phi_{i}=\int_{D_{i}}^{D_{i}+\Delta D}\frac{1}{\sqrt{2\pi }\sigma }e^{-\frac{{\left(D^{\prime }-D\right)}^{2}}{2\sigma^{2}}}dD^{\prime }\approx \frac{\Delta D}{\sqrt{2\pi }\sigma }e^{-\frac{{\left(D_{i}-D\right)}^{2}}{2\sigma^{2}}}\\ D_{i}\in [D-3\sigma ,D+3\sigma ]\;D_{i}-3\sigma >0 \end{array}$$

(9) $$\lambda_{g}\approx \sum_{i=1}^{n}\left[\frac{\Delta D}{\sqrt{2\pi }\sigma }e^{-\frac{{\left(D_{i}-D\right)}^{2}}{2\sigma^{2}}}\right]\cdot K\left(D_{i}\right)\;\left(D_{i}>0\right)$$

where *n* represents the number of pore size, $\Phi_{i}$ represents the contribution of pore size to the total porosity, and $K\left(D_{i}\right)$ is the simplified form of Kaganer model with pore size $D_{i}$.

Pore size modification model 2: In order to predict the gas thermal conductivity of aerogel more accurately, Bi et al. [34] in 2012 considered that: (1) most pore sizes present random distribution characteristics; (2) The contribution of macropores to gas thermal conductivity should be emphasized; (3) The contribution of small pores to gas thermal conductivity should be reduced. Based on these considerations, Bi et al. proposed a new random pore size distribution model for nano-porous materials to consider the randomness and heterogeneity of pore distribution in aerogel materials.

Bi et al. [34] modified the gas thermal conductivity model based on Gauss distribution proposed by Rechenauer et al. [33]. The authors modified the pore size distribution in aerogel materials, and Equation (10) is used to describe the pore size distribution. Based on this, Formulas (7), (9) and (10) are called the Non-Uniform Pore-Size (NUPS) model.

(10) $$\Phi_{i}=\{\begin{array}{ll} \frac{\Delta D}{\sqrt{2\pi }\sigma }e^{-\frac{{\left(D_{i}-D\right)}^{2}}{2\sigma^{2}}} & D_{i}\in [D-\sigma ,D+\sigma ]\;D_{i}-\sigma >0\\ \frac{2\Delta D}{\sqrt{2\pi }\sigma }e^{-\frac{{\left(D_{i}-D\right)}^{2}}{2\sigma^{2}}} & D_{i}\in (D+\sigma ,D+3\sigma ] \end{array}$$

In the NUPS model, the confidence interval is changed from [D−3σ,D+3σ] to [D−σ,D+3σ], and the pore size distribution is assumed to obey the Gaussian distribution in the confidence interval [D−σ,D+σ], so 68.26% of the pores could be guaranteed to obey the Gaussian distribution. Once the pore is within the interval of [D+σ,D+3σ], the value of the probability density function will be doubled based on the original value of the Gaussian distribution function.

Secondly, the expression of another key parameter $l_{m}$ in the Kaganer model is derived in free space, and the concept of mean free path used only considers the collision between gas molecules. However, the pore size of aerogel is lower than the mean free path of gas molecules, and the collision between gas molecules and pore wall will greatly limit the collision between gas molecules, thus reducing the gas thermal conductivity. Based on the above reasons, Zeng et al. [35,36,37] modified the mean free path of gas molecules on the basis of Kaganer model.

Modification of the mean free path of gas molecules: Zeng et al. [35,36,37] used probability theory to derive the expression of mean collision frequency of gas molecules in nanopores:

(11) $$f=\frac{uS_{s}\rho_{\mathrm{por}}}{4\phi }+\sqrt{2}\pi uN_{g}d_{g}^{2}$$

where u is the average motion velocity of molecules, $S_{s}$ is the specific surface area, $\rho_{\mathrm{por}}$ is the apparent density, ϕ is the porosity, and $N_{g}$ is the number density of gas molecules.

In Equation (11), the first part and the second part are, respectively, considered to be the mean collision frequency between gas molecules and solid walls and the mean collision frequency between gas molecules. Through Equation (11), the calculation formula of the mean free path of gas molecules can be obtained:

(12) $$l_{m}=u/f=\frac{1}{0.25S_{s}\rho_{\mathrm{por}\;}/\phi +\sqrt{2}\pi N_{g}d_{g}^{2}}$$

On this basis, the gas thermal conductivity in the aerogel nano-porous material is shown in Equation (13):

(13) $$\lambda_{g}=\frac{60.22\times 10^{5}pT^{-0.5}}{0.25S_{s}\rho_{\mathrm{por}}/\phi +4.01\times 10^{9}pT^{-1}}$$

The calculation model of gas thermal conductivity derived from the molecular motion theory is in good agreement with the experimental data. However, to accurately predict the gas thermal conductivity of different materials under different working conditions, the theoretical model needs to be modified in combination with relevant experimental data.

#### 3.1.2. Calculation Model of Solid Thermal Conductivity

**Figure 8:**
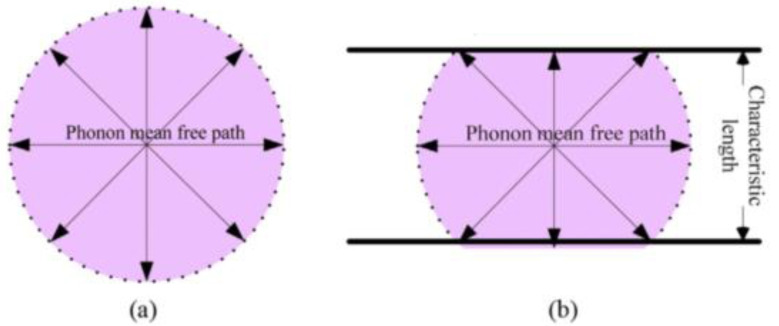
Diagram of size effect of solid thermal conductivity: (**a**) free space; (**b**) nanoscale heat transfer in a limited space.

Aerogel nano-porous material is a three-dimensional network skeleton structure formed by cross-linking nanoscale particles, which has been introduced in detail in the second chapter of heat transfer mechanism, and it has strong “path effect” and “nanoscale effect”, as shown in Figure 8 [4]. Similarly, for the summary of solid phase heat transfer model of aerogel materials, solid thermal conductivity is still divided into the empirical correlation formula based on experimental data, the numerical calculation method based on solving internal heat transfer equation, and the theoretical derivation model based on dynamics theory.

##### Empirical Correlation Formula

Since the pore size varies with the density of aerogel, the solid thermal conductivity of aerogel is fitted into the empirical correlation formula of material density based on the measurement results [28], as shown in Formula (14):

(14) $$\lambda_{s}\propto \rho_{a}^{1.5}$$

where $\lambda_{s}$ is the gas thermal conductivity and $\rho_{a}$ is the apparent density of the aerogel.

The disadvantage of the empirical correlation formula shown in Equation (14) is that it lacks strict theoretical basis. The calculation results of solid thermal conductivity are only affected by macroscopic density and cannot reflect the influence of the microscopic solid skeleton structure and pore size on solid thermal conductivity.

##### Numerical Simulation Method

The numerical simulation method for heat conduction of nanoscale solid materials is mainly based on solving the Boltzmann transport equation, as shown in Formula (2). By solving the Boltzmann transport equation, Chen et al. [38] found that when the particle diameter of the solid is much larger than the phonon mean free path of the solid, the heat transfer behavior of the solid is consistent with that under macroscopic conditions, satisfying Fourier’s heat conduction law. However, when the particle diameter is close to or less than the phonon mean free path, the heat transfer rate of the particle decreases significantly, resulting in the temperature rise of the particle far below the value predicted by Fourier’s heat conduction law. According to the research results, Chen et al. [38] proposed an approximate formula for calculating the thermal conductivity of nanoparticles:

(15) $$\lambda_{s}=\frac{3r^{*}/4}{3r^{*}/4+1}\lambda_{\mathrm{bulk}\;}$$

where $r^{*}=r/\Lambda$ is the dimensionless radius of solid particles, *r* is the radius of solid particles, Λ is the phonon mean free path of solid, and $\lambda_{\mathrm{bulk}}$ is the thermal conductivity of aerogel bulk materials.

Wang et al. [39], Zhao et al. [40], Xie et al. [41] and Han et al. [42] all used Equation (15) to modify the thermal conductivity of nanoscale solid skeleton particles.

##### Theoretical Derivation Method

Aerogel solid phase structure is complex and usually presents an amorphous state. The thermal conductivity of amorphous solid mainly depends on the local vibration of solid atoms/phonons and is limited by the scattering of atoms/phonons at the mean collision distance (mean free path). Based on this, Cahill et al. [43,44] first proposed the theory of minimum thermal conductivity to predict the thermal conductivity of amorphous solids:

(16) $$\lambda_{\min}={\left(\pi /6\right)}^{1/3}k_{B}n^{2/3}\sum_{i}v_{i}{\left(T/\Theta_{i}\right)}^{2}\int_{0}^{\Theta_{i}/T}\frac{x^{3}e^{x}}{\left(e^{x}-1\right)}dx$$

where *n* is the atomic density, *v* is the sound speed of solid, *i* represents the three acoustic modes, and $\Theta_{i}$ is the cutoff frequency of each polarization.

Because it is complicated to calculate the solid thermal conductivity by Formula (16), it is not convenient for engineering application. Most scholars use Formula (17) to calculate the solid thermal conductivity in aerogel nano-porous materials [45,46].

(17) $$\lambda_{S}=\lambda_{0}\frac{\rho }{\rho_{0}}\frac{v}{v_{0}}$$

where ρ is the apparent density of aerogel, $\rho_{0}$ is the density of aerogel skeleton, *v* is the sound velocity in aerogel, *v*_0_ is the sound velocity of solid skeleton, and $\lambda_{0}$ is the thermal conductivity of aerogel skeleton (thermal conductivity of heat transfer along aerogel solid skeleton).

It is difficult to obtain the value of $\lambda_{0}$ in Equation (17), and $\lambda_{0}$ in the existing literature is generally not accurate. Based on this, Bi et al. [47,48,49,50] calculated the thermal conductivity of aerogel solid skeleton by the following formula according to the kinetic theory:

(18) $$\lambda_{0}=\frac{1}{3}C_{V}v_{0}\Lambda_{0}$$

where $C_{V}$ is the specific heat of volume and $\Lambda_{0}$ is the phonon mean free path.

#### 3.1.3. Calculation Model of Gas-Solid Coupling Thermal Conductivity

Reichenauer et al. [33] and Swimm et al. [51] both pointed out that gas-solid coupling heat transfer is an important heat transfer mode among aerogel heat transfer modes, but this heat transfer mode is ignored by most scholars. Several main calculation models of gas-solid coupling thermal conductivity are introduced below.

**Figure 9:**
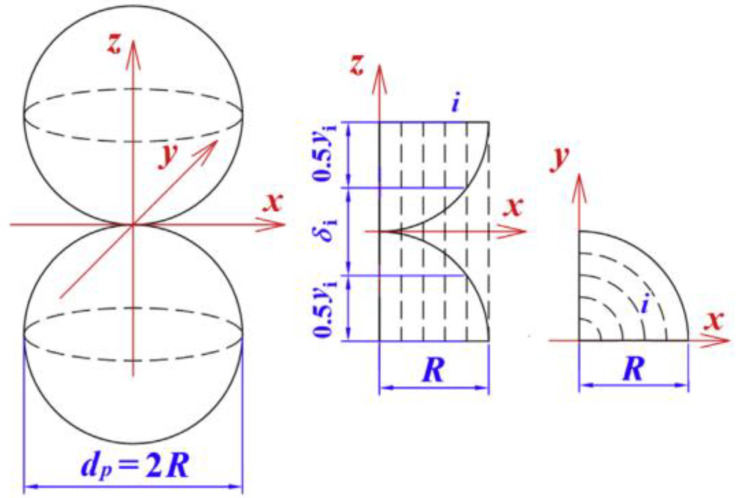
Gas-solid coupling thermal conductivity model established by Swimm et al. [52] (*R* is the radius of the particle).

Swimm et al. [52] experimentally studied the thermal insulation performance of RF (resorcin-formaldehyde) aerogel, as shown in Figure 9, and proposed a gas-solid coupling thermal conductivity model based on RF aerogel:

(19) $$\lambda_{c}=1.5(1-\Phi )\frac{2R}{\pi R^{2}}\sum_{i=1}^{n}{\left(\frac{\delta_{i}}{\lambda_{g,i}A_{i}}+\frac{y_{i}}{\lambda_{p}A_{i}}\right)}^{-1}$$

(20) $$y_{i}=2{\left[R^{2}-{\left(iR/n\right)}^{2}\right]}^{0.5}$$

(21) $$\delta_{i}=2R-y_{i}$$

(22) $$A_{i}=\pi (1+2i){\left(R/n\right)}^{2}$$

where *R* (*R* = 0.5$d_{p}$) is the particle diameter of the aerogel skeleton, *i* is the *i*th hollow cylinder in the cylinder unit, n represents the number of hollow cylinders, $y_{i}$ is the total height of the solid phase of the *i*th hollow cylinder, $\delta_{i}$ is the corresponding gap size, $A_{i}$ is the annular area of the *i*th hollow cylinder. In addition, $\lambda_{g,i}$ is the gas thermal conductivity with the gap size of $\delta_{i}$, $\lambda_{p}$ is the thermal conductivity of the solid particles in the aerogel skeleton.

**Figure 10:**
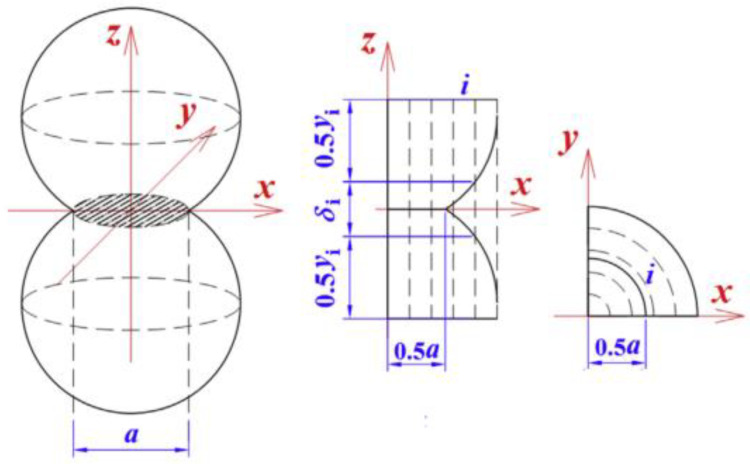
Gas-solid coupling thermal conductivity model established by Zhao et al. [25] (*a* and *i* represent the contact area of the particles and the *i*th hollow cylinders in the cylindrical unit cell, respectively).

Zhao et al. [25] pointed out that the connected solid particles in the aerogel skeleton have a certain contact diameter, rather than point contact, as shown in Figure 10. Therefore, Equation (19) is modified.

**Figure 11:**
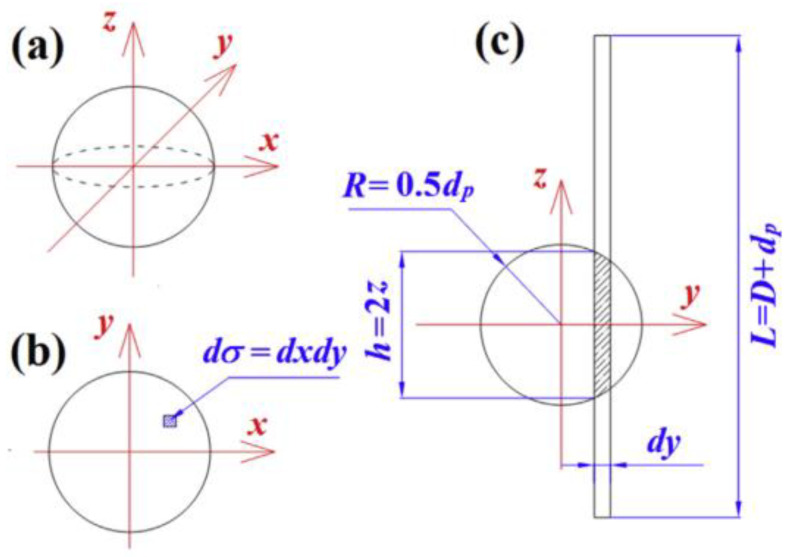
Gas-solid coupling thermal conductivity model established by Bi et al. [53] (h represents length of heat flux path in the solid phase).

The gas-solid coupling heat transfer calculation models derived by Swimm et al. and Zhao et al. involve a large number of parameters and require numerical integration, which bring difficulty and inconvenience to calculation and engineering application. Therefore, Bi et al. [53] established a new gas-solid coupling heat transfer model (as shown in Figure 11, which can be expressed as:

(23) $$\lambda_{c}=\frac{D+d_{p}}{d_{p}}\frac{2\lambda_{p}\lambda_{g}}{\lambda_{g}-\lambda_{p}}[1-\frac{D+d_{p}}{d_{p}}\frac{\lambda_{p}}{\lambda_{g}-\lambda_{p}}\ln\times \left(1+\frac{\lambda_{g}-\lambda_{p}}{\lambda_{p}}\frac{d_{p}}{D+d_{p}}\right)]$$

**Figure 12:**
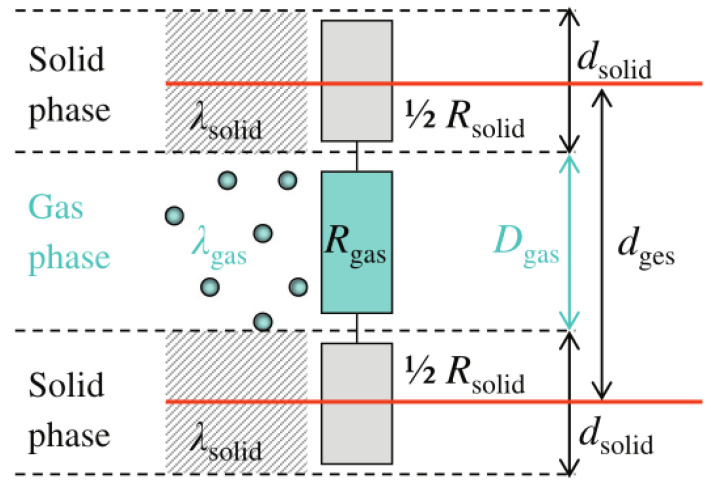
Resistance model [54].

In 2017, Swimm et al. [54] pointed out that the accuracy of existing calculation models of gas-solid coupling thermal conductivity is poor in consistency with the test results, the accuracy needs to be improved, and some models are too complex for practical application. Based on this, Swimm et al. analyzed and described the gas-solid coupling thermal conductivity through the series connection of gas and solid phase thermal resistance, as shown in Figure 12. The derivation of gas-solid coupling thermal conductivity can be simply described by the series connection of the thermal resistance $R_{\mathrm{gas}}$ and $R_{\mathrm{solid}}$, and the final derivation model of gas-solid coupling thermal conductivity is shown in Formula (24):

(24) $$\begin{array}{l} \lambda_{c}\left(p_{g},T\right)={\left(R_{\mathrm{solid}\;}+R_{\mathrm{gas}\;}\right)}^{-1}\cdot d_{\mathrm{ges}\;}\\ ={\left[\frac{d_{\mathrm{solid}\;}}{\lambda_{\mathrm{solid}\;}(T)}+\frac{D_{\mathrm{gas}\;}}{\lambda_{\mathrm{gas}\;}\left(p_{g},T\right)}\right]}^{-1}\cdot \left(d_{\mathrm{solid}\;}+D_{\mathrm{gas}\;}\right)\\ =\lambda_{\mathrm{gas}\;}\left(p_{g},T\right)\cdot \frac{\lambda_{\mathrm{solid}\;}(T)}{\lambda_{\mathrm{gas}\;}\left(p_{g},T\right)\cdot d_{\mathrm{solid}\;}+\lambda_{\mathrm{solid}\;}(T)\cdot D_{\mathrm{gas}\;}}\cdot \left(d_{\mathrm{solid}\;}+D_{\mathrm{gas}\;}\right)\\ \equiv \lambda_{\mathrm{gas}\;}\left(p_{g},T\right)\cdot f\left(p_{g},T\right). \end{array}$$

To sum up, it can be known from the literatures that by considering only gas thermal conductivity, solid thermal conductivity, and radiation thermal conductivity, the predicted results are mostly lower than the experimental data, which are usually attributed to the coupled heat transfer between gas molecules and aerogel particles. In view of this, domestic and foreign scholars have conducted some researches on gas-solid coupling heat transfer, but there are still few researches on this kind of study.

#### 3.1.4. Calculation Model of Radiation Thermal Conductivity

Aerogel material is a participating medium for radiation heat transfer, and the radiation heat transfer inside the material belongs to medium radiation. When radiation heat is injected into the material, the material will produce absorption and scattering effects on radiation, which shows that the material has attenuation effects on radiation [55]. At normal temperature, the radiation heat transfer in the medium may not be significant, but at high temperature, the radiation heat transfer cannot be ignored. It must be mentioned that aerogel has strong permeability to near-infrared radiation with wavelengths of 3–8 μm at high temperature, which leads to poor shielding ability of aerogel at high temperature, and the thermal conductivity of aerogel increases significantly with the increase in temperature [56,57].

**Figure 13:**
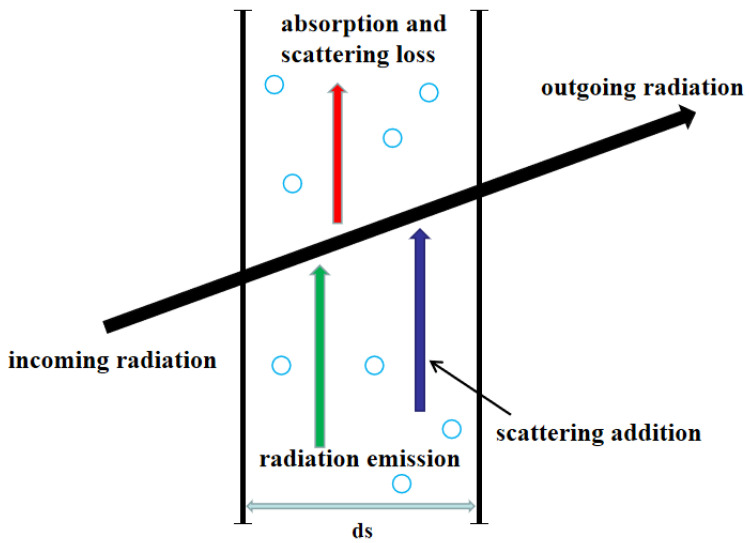
Schematic diagram of radiation heat transfer.

As shown in Figure 13, it is the radiation heat transfer process inside the aerogel medium. The radiative intensity within aerogel is governed by the radiative transport equation (RTE) [58], and the RTE is shown in Formula (25).

(25) $$\frac{dI_{\lambda }(r,s)}{ds}=-\beta_{\lambda }I_{\lambda }(r,s)+\kappa_{\lambda }I_{b\lambda }(r)+\frac{\sigma_{s\lambda }}{4\pi }\int_{\Omega_{i}=4\pi }I_{\lambda }(r,s_{i})\Phi_{\lambda }\left(s_{i},s\right)d\Omega_{i}$$

where β, κ, $\sigma_{s}$ are the extinction, absorption and scattering coefficients, respectively; $I_{b}(r)$ is the radiative intensity emitted by a black body; I(r,s) represents the radiative intensity of space position r and transmission direction s, which is a vector; $\Phi \left(s_{i},s\right)$ is the scattering phase function, which is the ratio of the scattering intensity in the **s** direction caused by incident radiation in the $s_{i}$ direction to the average scattering intensity in the 4π scattering space. Here, because the RTE is related to space and direction, I(r,s), $I(r,s_{i})$ and $\Phi \left(s_{i},s\right)$ in the Equation (9) are all related to direction, which are vectors.

For aerogel nano-porous materials, since most of the existing aerogel materials are optically thick medium, the Rosseland formula is the most widely used formula in domestic and foreign research and engineering calculation. For optically thick media, the optical thickness (the product of the attenuation coefficient and the characteristic thickness of the material) is much greater than 1. If the medium is optically thick, the attenuation effect of the medium on radiation is strong, resulting in a very short transfer distance of radiation energy, so the energy transfer characteristics are similar to that of solid heat conduction. Thus, the RTE can be simplified, and the simplified RTE is called the optical thickness approximation formula, also known as the Rosseland formula [59].

According to the Rosseland formula, radiation heat flux can be expressed by the following formula:

(26) $$q_{r}(x)=-\frac{16}{3\sigma_{e,R}}n^{2}\sigma T^{3}\frac{\partial T}{\partial x}=-\lambda_{r}\frac{\partial T}{\partial x}$$

(27) $$\lambda_{r}=\frac{16}{3\sigma_{e,R}}n^{2}\sigma T^{3}$$

where *T* is the temperature, *n* is the average refraction index, *σ* is the Stefan–Boltzmann constant, $\sigma_{e,R}$ is the mean attenuation coefficient, and $\lambda_{r}$ is the radiation thermal conductivity.

Due to the simple form of the Rosseland formula, and the expression of radiation heat flux is similar to Fourier’s law of heat conductivity, the Rosseland formula is widely used by most scholars, such as Lu et al. [60,61], Lee et al. [62], Zhang et al. [63], Soorbaghi et al. [64] and Dai et al. [65].

### 3.2. Calculation Models of Effective Thermal Conductivity

The effective thermal conductivity of aerogel represents the overall thermal insulation performance of the material. Different heat transfer modes in aerogels are analyzed in order to obtain the overall thermal conductivity of aerogels and provide theoretical basis for the design of thermal insulation materials. The common methods for calculating the effective thermal conductivity of aerogel include the decoupling method, the equivalent circuit method and the numerical simulation method. Before introducing the three methods in detail, the disadvantages and advantages of the three methods are briefly summarized in Table 2.

#### 3.2.1. Decoupling Method

In the decoupling model, many scholars at home and abroad assume that the three heat transfer modes of nano-porous materials, namely gas heat transfer, solid heat transfer and radiation heat transfer, are independent of each other, so the effective thermal conductivity of nano-porous materials is the sum of the three heat transfer modes, as shown in Equation (28):

(28) $$\lambda_{\mathrm{eff}}=\lambda_{S}+\lambda_{g}+\lambda_{r}$$

However, according to [33,51], most of the predicted results obtained by Equation (28) are lower than the experimental data, which are usually attributed to the coupled heat transfer between gas molecules and aerogel particles (coupling effect). In order to consider the contribution of coupling effect to effective thermal conductivity, [25,52,53,54] point out that gas-solid coupling heat transfer is the fourth heat transfer mode in aerogel, and Formula (28) is modified as follows:

(29) $$\lambda_{\mathrm{eff}}=\lambda_{S}+\lambda_{g}+\lambda_{r}+\lambda_{c}$$

#### 3.2.2. Equivalent Circuit Method

An important method to establish the theoretical calculation model of the effective thermal conductivity of aerogel materials is to obtain the corresponding calculation model by the equivalent circuit method for a certain structure.

**Figure 14:**
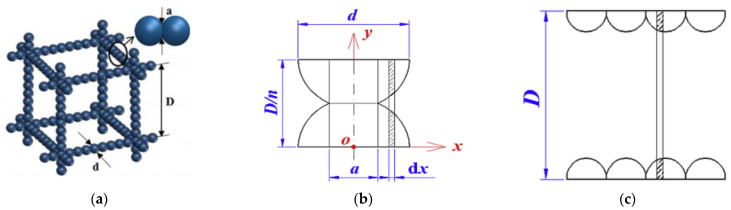
Cross ball rod structure: (**a**) Cross ball rod structure; (**b**) Structure diagram of $q_{s}$ and $q_{\mathrm{sg}}$; (**c**) Structure diagram of $q_{\mathrm{sgs}}$.

When Zeng et al. [66] studied the overall thermal conductivity of aerogel materials, three typical structural units were used to characterize the aerogel nano-porous materials, namely, the cross square rod structure, the cross cylindrical rod structure, and the cross ball rod structure. Under the assumption of one-dimensional heat transfer, the equivalent circuit method is used to calculate the effective thermal conductivity of aerogel under three conditions. The structure of cross ball rod is shown in Figure 14, and the sum of gas thermal conductivity, solid thermal conductivity, and gas-solid coupling thermal conductivity is shown in Equation (30):

(30) $$\begin{array}{l} \lambda_{g}+\lambda_{s}+\lambda_{c}=\frac{q_{g}+q_{s}+q_{\mathrm{sg}}+q_{\mathrm{sgs}}}{\Delta TD}=D^{2}\{\frac{\pi \lambda_{g}d_{p}}{2nd_{p}\alpha }\left[-\sqrt{1-\beta^{2}}-\frac{D}{nd_{p}\alpha }\ln\left(1-\frac{nd_{p}\alpha }{D}\sqrt{1-\beta^{2}}\right)\right]\\ +\frac{a\lambda_{s}}{1.1nd_{p}}+\frac{(n-1)\pi \lambda_{g}d_{p}}{D\alpha }\left(\beta -1+\frac{D}{\alpha d_{p}}\ln\frac{D-\alpha a}{D-\alpha d}\right)\\ +{\left(1-d_{p}/D\right)}^{2}\lambda_{g}\}\frac{\Delta T}{D} \end{array}$$

where $q_{g}$, $q_{s}$, $q_{\mathrm{sg}}$ and $q_{\mathrm{sgs}}$ represent the heat transferred through the gas phase, the heat transferred through the solid phase, the heat transferred by the gas in the gap between the two spheres, and the heat transferred from the bottom sphere to the top sphere through the gas phase respectively, and ΔT represents the temperature difference between the bottom and the top of the unit.

Based on the Zeng model, Wei et al. [67] theoretically derived the effective thermal conductivity of aerogel composite insulation material by using the equivalent circuit method, as shown in Equation (31):

(31) $$\begin{array}{l} \lambda_{g}+\lambda_{s}+\lambda_{c}=\{\frac{\left(2-\gamma_{b}\right)\gamma_{a}^{2}\gamma_{b}}{1-\beta_{1}\gamma_{a}}+\frac{\gamma_{a}^{2}\left[{\left(1-\gamma_{b}\right)}^{2}-\gamma_{c}^{2}\right]}{1-\beta_{1}\gamma_{a}\gamma_{b}}+\frac{2\gamma_{a}\gamma_{c}\left(1-\gamma_{a}\right)}{1-\beta_{1}\gamma_{a}\gamma_{c}}\\ +\frac{\gamma_{a}^{2}\gamma_{c}^{2}}{1-\beta_{1}+\beta_{1}\left(1-\gamma_{b}\right)\gamma_{a}}+\left(1-\gamma_{a}\right)\left(1+\gamma_{a}-2\gamma_{a}\gamma_{c}\right)\}\\ \cdot \left[\psi k_{\mathrm{ae}}+(1-\psi )k_{g}\right]\;(0<c<a-2h)\\ \lambda_{g}+\lambda_{s}+\lambda_{c}=\{1+\frac{2\gamma_{a}\gamma_{c}\left(1-\gamma_{a}\right)}{1-\beta_{1}\gamma_{a}\gamma_{c}}+\frac{{\left(1-\gamma_{b}\right)}^{2}\gamma_{a}^{2}}{\gamma_{a}\left(1-\gamma_{b}\right)+\left(1-\gamma_{a}+\gamma_{a}\gamma_{b}\right)\left(1-\beta_{1}\right)}\\ +\frac{\left(1-\gamma_{c}^{2}\right)\gamma_{a}^{2}}{1-\beta_{1}\gamma_{a}}+\frac{\gamma_{a}^{2}\left[\gamma_{c}^{2}-{\left(1-\gamma_{b}\right)}^{2}\right]}{1-\beta_{1}}+\gamma_{a}\left(1-\gamma_{a}-2\gamma_{c}+\gamma_{a}\gamma_{c}\right)\}\\ \cdot \left[\psi k_{\mathrm{ae}}+(1-\psi )k_{g}\right]\;(a>c>a-2h) \end{array}$$

**Figure 15:**
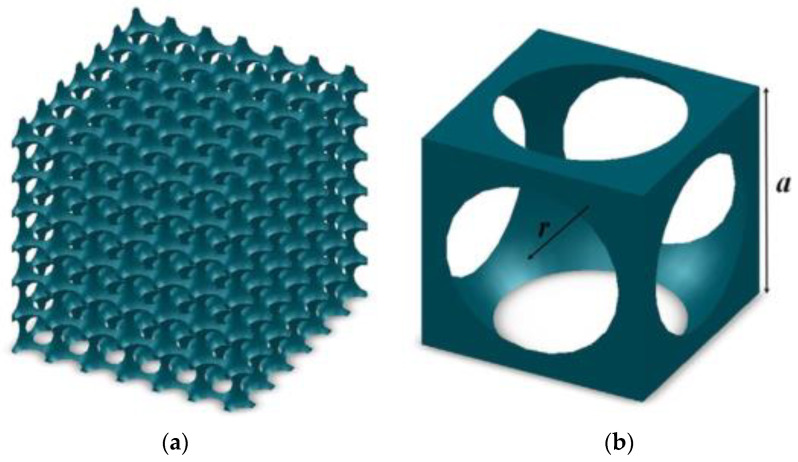
Spherical hollow cube model: (**a**) Overall view; (**b**) A spherical hollow cube.

**Figure 16:**
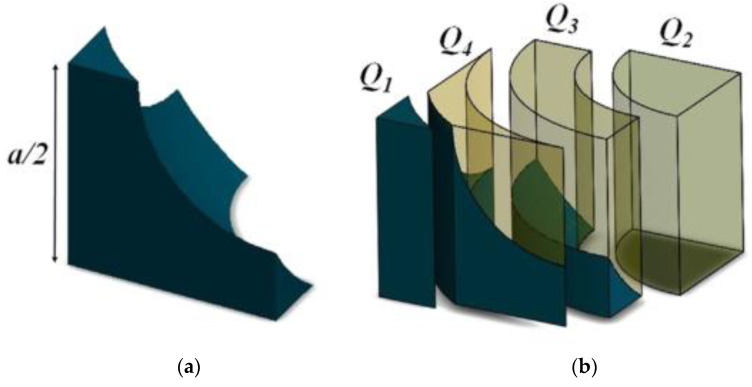
Cell: (**a**) Cell; (**b**) One-dimensional heat transfer.

Dan et al. [68], based on the structure of aerogel and Zeng model, proposed a new model, namely the spherical hollow cube model. The spherical hollow cube model and cell are shown in Figure 15 and Figure 16, respectively. The calculation formula of the sum of gas phase thermal conductivity, solid phase thermal conductivity, and gas-solid coupling thermal conductivity is shown in Equation (32):

(32) $$\begin{array}{l} \lambda_{g}+\lambda_{s}+\lambda_{c}=\frac{Q_{1}+Q_{2}+Q_{3}+Q_{4}}{\Delta T\cdot a/2}=\frac{2\lambda_{s}\left[\frac{a^{2}-\pi r^{2}}{2}+2\arccos\left(\frac{a}{2r}\right)r^{2}-a\sqrt{r^{2}-{\left(\frac{a}{2}\right)}^{2}}\right]}{a^{2}}+\lambda_{g}\pi \left(\frac{r^{2}}{a^{2}}-\frac{1}{4}\right)\\ -\frac{\lambda_{s}\pi }{k^{2}a}[kr\left(\cos\theta_{1}-\cos\theta_{0}\right)-\frac{a}{2}\ln\left(\frac{kr\cos\theta_{1}+a/2}{kr\cos\theta_{0}+a/2}\right)]+\frac{4}{a}\int_{a/2}^{r}\frac{\arcsin\left(\frac{a/2-\sqrt{x^{2}-a^{2}/4}}{\sqrt{2}x}\right)xdx}{\sqrt{r^{2}-x^{2}}/\lambda g+\left(a/2-\sqrt{r^{2}-x^{2}}\right)/\lambda_{s}} \end{array}$$

The thermal conductivity model of thermal insulation composites filled with aerogel particles was proposed by He et al. [69]. The corresponding models were established and the thermal conductivity equations based on the analysis of the heat transfer in the composite are derived in this paper.

**Figure 17:**
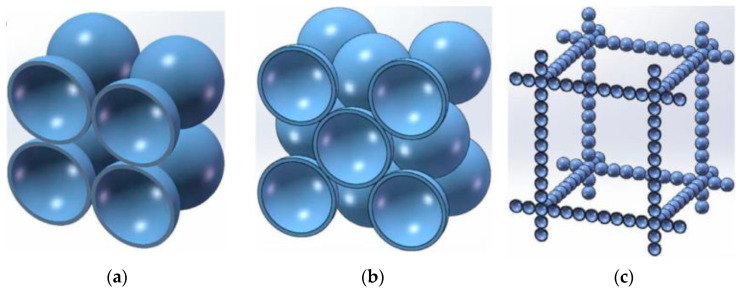
Regular geometric structure: (**a**) Cubic packing structure; (**b**) The face-centered cubic packing structure; (**c**) The cubic array of intersecting spheres.

In Liu’s study [70], three kinds of nano-porous insulating materials with regular geometric structures and controllable thermal conductivities, including a simple cubic packing, a face-centered cubic packing, and a cubic array of intersecting spheres packing of uniform-sized hollow nanospheres, were designed, as shown in Figure 17. The effective thermal conductivity models of each packing structure are developed according to the assumption of one-dimensional heat transfer, in which the following factors including material types, size of the hollow nanosphere packing structure (e.g., sphere size, spherical shell thickness, contact ratio), gas pressure, the rarefaction effect of gas and the mean free path of phonons were considered.

Similar to Zeng et al., Wei et al., Dan et al., He et al., and Dan et al., in the research of many domestic and foreign scholars [71,72], the cross ball rod structure has also been used to characterize the nano-porous structure of aerogel matrix materials. In fact, most studies on equivalent circuit method use some regular structures to characterize the nano-porous structure of aerogel materials, while the complex nano-porous structure of aerogel materials is ignored. In order to make the calculation results more accurate, Xie et al. [41], Pia et al. [73], Li et al. [74] and Chen et al. [75] all adopted the fractal structure to analyze the microstructure of aerogel materials, which will not be described in detail here.

#### 3.2.3. Numerical Simulation Method

The prediction of the effective thermal conductivity of aerogel by the decoupling method and equivalent circuit method cannot provide much information about the heat transfer in aerogel, such as temperature distribution and heat flux distribution. Therefore, in order to further study the heat transfer mechanism in aerogel nano-porous materials, numerical simulation based on solving the energy equation inside the material is used to determine the temperature distribution and heat flux field in the nano-porous materials. The energy equations of heat transfer in the material are shown in Equations (33) and (34), respectively. The energy equation considering only heat conduction is shown in Equation (33). If the radiation heat transfer in the medium is taken into account, Equations (34) and (25) mentioned in the previous section should be solved simultaneously to realize the coupling solution of thermal conductivity and radiation heat transfer.

(33) $$\rho c\frac{\partial T(x,y,z)}{\partial t}=-\nabla \cdot q_{t}=-\nabla \cdot q_{c}=\frac{\partial }{\partial x}\left(\lambda \frac{\partial T}{\partial x}\right)+\frac{\partial }{\partial y}\left(\lambda \frac{\partial T}{\partial y}\right)+\frac{\partial }{\partial z}\left(\lambda \frac{\partial T}{\partial z}\right)$$

(34) $$\rho c\frac{\partial T(x,y,z)}{\partial t}=-\nabla \cdot q_{t}=-\nabla \cdot q_{c}-\nabla \cdot q_{r}=\frac{\partial }{\partial x}\left(\lambda \frac{\partial T}{\partial x}\right)+\frac{\partial }{\partial y}\left(\lambda \frac{\partial T}{\partial y}\right)+\frac{\partial }{\partial z}\left(\lambda \frac{\partial T}{\partial z}\right)-\nabla \cdot q_{r}$$

**Figure 18:**
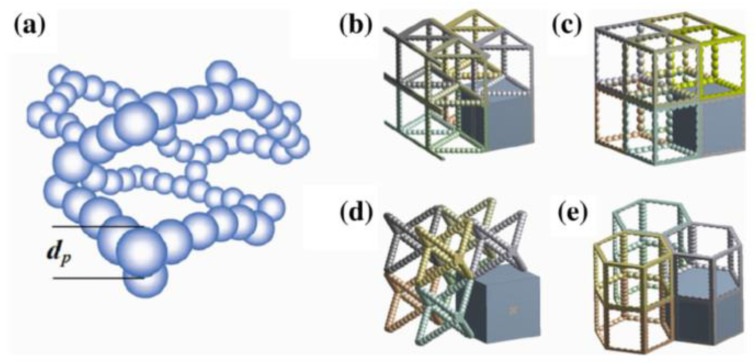
Schematic of base-catalyzed aerogel structures: (**a**) Aerogel backbone; (**b**) Regular triangular prism; (**c**) Cube; (**d**) Octahedron; (**e**) Regular hexagonal prism.

Before numerical calculation, the structure of the aerogel material needs to be established for numerical simulation. Aiming at the structural characteristics of aerogels nano-porous materials, Spagnol et al. [76] established a numerical model based on two-dimensional fractal structure to simulate heat transfer in silica aerogels. Zhao et al. [40] adopted the diffusion-limited cluster–cluster aggregation (DLCA) method to generate three-dimensional random particle stacking structure to characterize the typical structure of aerogel nano-porous materials, and then calculated the thermal conductively radiation coupling thermal conductivity of the materials by numerical simulation. Bi et al. [77] also adopted a similar approach to calculate the effective thermal conductivity of aerogel nano-porous materials. Four three-dimensional regular structures were used to represent the typical structures of aerogel nano-porous materials, namely, the regular triangular prism structure, cube structure, octahedron structure, and regular hexagonal prism structure, as shown in Figure 18.

**Figure 19:**
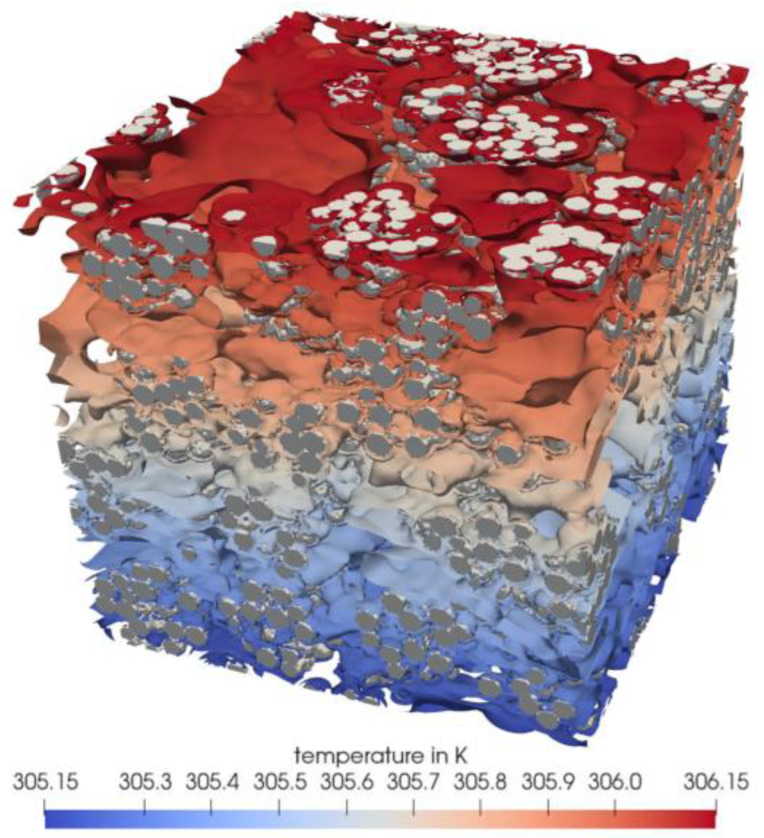
Temperature distribution simulated through three-dimensional packing of silica particles.

In recent years, Fang et al. [78], Han et al. [79], Kan et al. [80], Qu et al. [9] and Ross-Jones et al. [81] used LBM to simulate the heat transfer of nano-porous geometric structures, so that not only the effective thermal conductivity, but also the temperature distribution could be calculated. The temperature distribution calculated by Ross-Jones et al. [81] is shown in Figure 19.

## 4. Test Methods of Thermal Insulation Performance

In order to verify the accuracy of the thermal conductivity calculation model of aerogel nano-porous materials, correct experimental data are required to modify the model. The existing test methods are mainly divided into the heat conduction heating test method, infrared radiation heating test method, and convection heating test method [82]. Among the thermal conductivity test methods, the hot wire method, hot strip method, and transient plane source method of the unsteady state methods are widely used. However, Zhang et al. [83,84,85] pointed out that the unsteady state methods still have a large error when they are used to measure the thermal conductivity of aerogel nano-porous materials. For the infrared radiation heating, compared with the convection heating method, the infrared radiation heating method will have some extra energy penetrating into the material at high temperature, which enhances the degree of radiation heat transfer inside the material, so that the test result of the thermal conductivity of aerogel is larger. Before introducing the three methods in detail, the disadvantages and advantages of the three methods are briefly summarized in Table 3.

### 4.1. Heat Conduction Heating Method

At present, heat conductivity heating methods can be divided into two categories: the steady state method, in which the sample temperature distribution does not change with time, and the unsteady state method, in which the temperature distribution changes with time.

#### 4.1.1. Steady State Method

According to the second law of thermodynamics, heat will spontaneously flow from a body with a high temperature to a body with a low temperature. The main way of heat transfer in heat conduction heating method is heat conduction [86]. In the test sample, the internal temperature field is generally shown as Equation (35):

(35) T=f(x,y,z,τ)

where T represents temperature, *x*, *y* and *z* represent corresponding axes, respectively, and τ represents time.

The steady state method mainly includes the hot plate method and heat flux meter method. In the steady state method, the temperature field within the sample is a steady state temperature field, that is, the temperature does not change with time. In the sample, there is only one-dimensional heat transfer, so the mathematical expression of temperature field can be simplified as shown in Equation (36):

(36) T=f(y)

For one-dimensional steady state heat transfer, heat is transferred along the direction of temperature gradient. Fourier’s law of heat conduction can be used to calculate the effective thermal conductivity of the materials, as shown in Equation (37):

(37) $$\lambda =\frac{Q\times d}{A(T_{2}-T_{1})}$$

where *Q* is the total heat flux, *d* is the thickness of the sample, *A* is the area, $T_{2}$ and $T_{1}$ are the mean temperature of the hot side and cold side of the sample, respectively.

#### 4.1.2. Unsteady State Method

The reliability and accuracy of steady state method are ideal, but the long measurement period is considered to be the most outstanding feature. Especially when the thermal physical properties of insulation materials are measured, the size of the sample is generally required to be large enough to reduce the influence of the surrounding environment [58,87]. Because the thermal conductivity of thermal insulation materials is very low, it will take a long time to establish a stable temperature gradient in the thickness direction of the sample, which is more obvious when measuring at high temperature. Based on this, the unsteady state method is widely used to measure the thermal conductivity of thermal insulation materials, which is based on the unsteady state thermal conduction differential equation [88]. The measurement principle is to use a contact heat source to apply a trace amount of thermal interference to the sample, at the same time measure the temperature response of a point in the sample to the thermal interference, and then the thermal conductivity of the material is calculated according to the measured data. The unsteady state method mainly includes the hot wire method, hot strip method, transient plane heat source method and so on, among which the hot wire method is a the commonly used international standardized method for measuring thermal conductivity [89].

At present, the unsteady state method is used to measure the thermal conductivity of aerogel nano-porous materials by most domestic and foreign scholars. However, Zhang et al. pointed out that such measurement methods have many limitations when measuring the thermal conductivity of nano-porous materials; especially when the boundary temperature is high and the material has a low attenuation coefficient, the measurement error is more obvious. As the hot wire method is a commonly used method, the influence of thermal radiation on the measurement of nano-porous insulation materials by the hot wire method is mainly introduced here.

**Figure 20:**
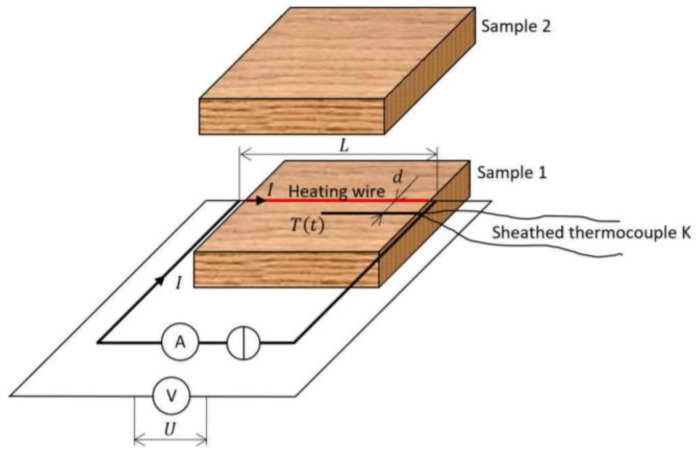
Schematic diagram of hot wire method.

The schematic diagram of hot wire method is shown in Figure 20 [90]. The basic principle is to place a heating wire, namely a hot wire, in the middle of the sample with uniform and isotropic temperature to be tested. According to the variation of sample temperature over time, the thermal conductivity of the sample can be solved, as shown in Equation (38):

(38) $$\lambda =\frac{Qd(\ln\tau )}{4\pi d(\theta (\tau ))}$$

where λ is the thermal conductivity of the sample to be measured, *Q* is the heating power of the hot wire per unit length, τ is the heating time of the hot wire, and θ(τ) is the temperature rise of the hot wire at τ time.

Zhang et al. pointed out that when the unsteady state methods were used to measure the thermal conductivity of nano-porous materials, there was a problem of inconsistency between the test theory and the actual heat transfer process. The influence of thermal radiation on effective thermal conductivity of nano-porous thermal insulation materials measured by hot wire method and transient plane source method is studied numerically.

**Figure 21:**
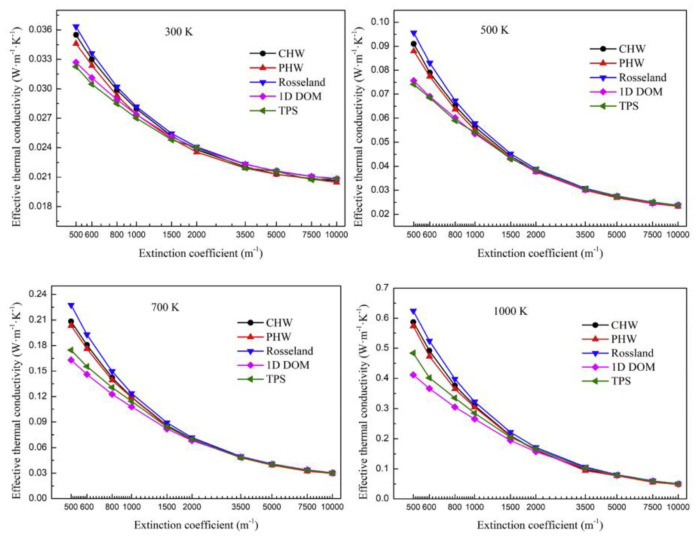
Influence of extinction coefficient.

**Figure 22:**
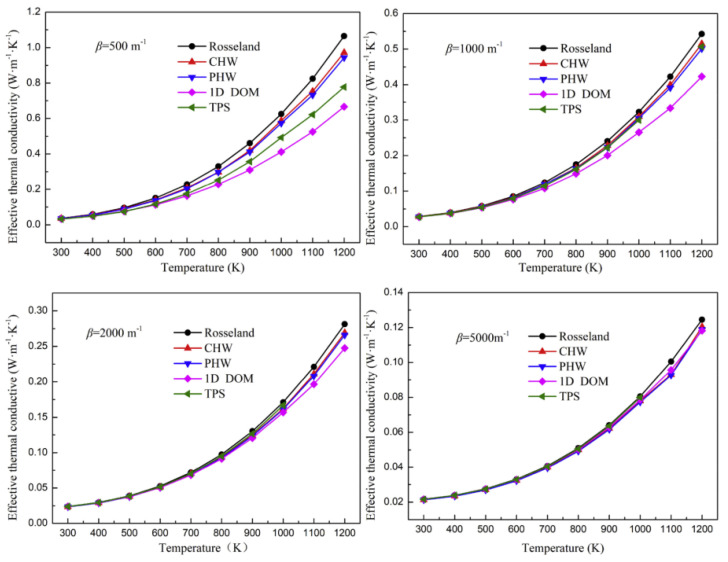
Influence of temperature.

The thermal conductivity measured by cross arrangement and parallel arrangement of hot wire method is written as CHW and PHW, respectively. The thermal conductivity measured by the transient plane source method is written as TPS. The thermal conductivity calculated by the Rosseland formula is written as Rosseland. The thermal conductivity obtained through the one-dimensional steady state simulation of the coupled heat transfer process of conduction and radiation is written as 1D DOM, which is the correct effective thermal conductivity of the material. The simulation results of the thermal conductivity change with the extinction coefficient and temperature, as shown in Figure 21 and Figure 22, respectively. The results show that the error of thermal conductivity measured by the unsteady state method decreases with the increase in the extinction coefficient, which is because the suppression of thermal radiation is enhanced. Temperature has the opposite effect on the measurement error, which is because the higher the temperature, the more obvious the thermal radiation.

### 4.2. Infrared Radiation Heating Method

Aerogel nano-porous materials have strong permeability to near-infrared radiation with wavelengths of 3–8 μm at high temperature, which leads to the poor shielding ability of aerogel at high temperature, and the thermal conductivity of aerogel increases significantly with the increase in temperature [24]. For this reason, compared to the heat conduction heating and convective heating methods for measuring nano-porous materials, the infrared heating method has “extra” energy penetrating into the aerogels, resulting in higher measurement results.

**Figure 23:**
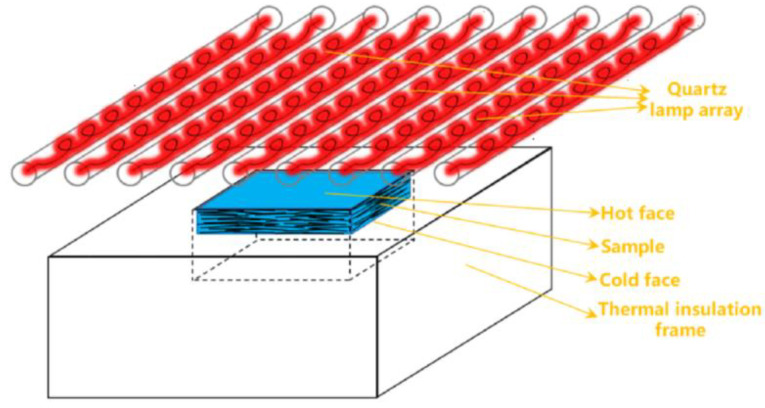
Schematic diagram of the quartz lamp heating test.

Peng et al. [91] developed a mullite fiber-reinforced alumina-silica aerogel composite, which was heated by a quartz lamp at 1500 °C to verify thermal insulation performance, as shown in Figure 23. At the same time, Liu et al. [92] also used a quartz lamp heating test to verify the thermal insulation performance of aerogel composites.

### 4.3. Convection Heating Method

**Figure 24:**
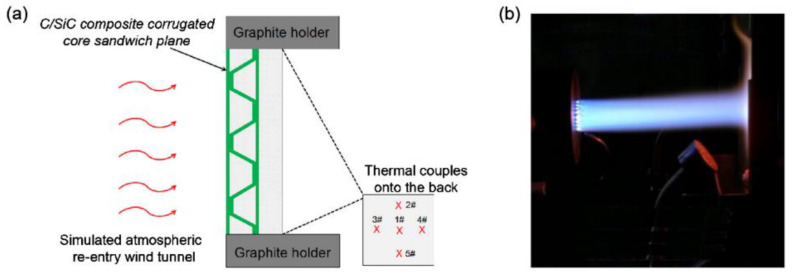
Combustion-gas wind tunnel test: (**a**) Arrangement of temperature thermocouple; (**b**) Atmospheric re-entry wind tunnel test.

The convective heating test method generally refers to the combustion-gas wind tunnel test, through which the real environment can be simulated. Li et al. [93] designed a new integrated thermal protection system. The inner core layer of the system was filled with insulating aerogel, and the thermal conductivity was obtained through the combustion-gas wind tunnel test, as shown in Figure 24.

**Figure 25:**
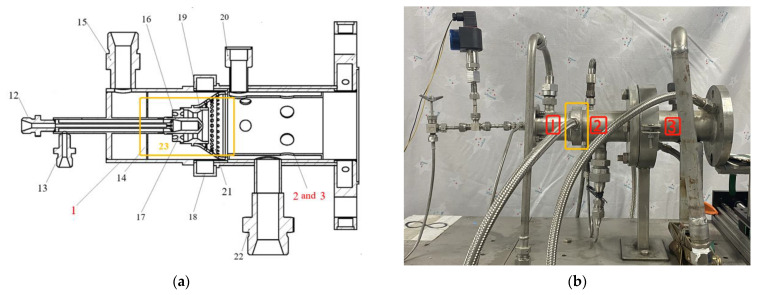
The combustion-gas generator: (**a**) Structure diagram; (**b**) Actual picture. 1, Air equalizing chamber; 2, The primary combustor; 3, The secondary combustor/mixer; 12, Kerosene inlet; 13, Atomization air inlet (the first stage atomization); 14, Uniform flow orifice; 15, The primary air inlet; 16, Atomization nozzle; 17, Air swirler; 18, Liquid trap; 19, Flame stabilizer; 20, Igniter; 21, Air equalizing cone; 22, The secondary air inlet; 23, The two-stage atomization device.

In order to simulate the real environment, the combustion-gas needs to have the advantages of high temperature, long working time, and fast transient change. However, there are still many shortcomings in the research on this aspect. In view of these problems, Lou et al. [94], Li et al. [95], and many domestic and foreign scholars have conducted a great deal of research. As shown in Figure 25, Lou et al. designed a combustion-gas wind tunnel device based on two-stage atomization, two-stage combustion/mixing mode of high temperature rise combustor. The ignition reliability, combustion stability, outer wall temperature of the combustor, combustion efficiency, outlet temperature distribution coefficient, radial temperature distribution coefficient, and total pressure recovery coefficient of the device were all within a reasonable range, and the most important point is that the combustion-gas wind tunnel device can achieve continuous, accurate, rapid and ultra-wide linear adjustment of the combustion-gas temperature.

## 5. Summary and Outlook

There are three models for predicting the effective thermal conductivity of aerogels nano-porous insulation materials. Firstly, mathematical models of different heat transfer modes are added in the decoupling method, which is simple in form and convenient in processing, and has been calculated by many scholars at home and abroad for the effective thermal conductivity. Secondly, in the study of the equivalent circuit method, some regular structures are used to characterize the nano-porous structures of aerogel materials. These regular structures simplify the heat transfer analysis of aerogel materials, but the complex nano-porous structure of aerogel materials is ignored. For the third method, the numerical simulation method is used to calculate heat transfer process within the material based on specific structures and certain boundary and initial conditions. The method is accurate enough to take into account the influence of various factors on heat transfer. However, the disadvantage is that the calculation is complicated and only discrete numerical results can be obtained. It must be mentioned that no matter what method is used to predict the effective thermal conductivity of aerogel nano-porous materials, the mathematical model needs to be modified for the effective thermal conductivity of different aerogel materials under different working conditions, combined with relevant experimental data.

In order to verify the accuracy of the thermal conductivity calculation model of aerogel nano-porous materials, correct experimental data are required to modify the model. The existing test methods are mainly divided into the heat conduction heating test method, the infrared radiation heating test method, and the convection heating test method. Among the thermal conductivity test methods, the hot wire method, the hot strip method, and the transient plane source method of the unsteady state methods are widely used. However, due to the heat transfer theory inside the material being inconsistent with the measurement principle of the device, the unsteady state methods still have a large error when they are used to measure the thermal conductivity of aerogel nano-porous materials. For the infrared radiation heating method, compared with the convection heating method, the infrared radiation heating method will have some extra energy penetrating into the material at high temperature, which enhances the degree of radiation heat transfer inside the material, so that the test result of the thermal conductivity of aerogel is larger.

In conclusion, although there is much literature that has studied the heat transfer characteristics of nano-porous silica aerogel insulation materials, some issues still need to be investigated to better reveal the heat transfer mechanism of the material: (1) Apply suitable methods to investigate the nanoscale effect, the interface effect, as well as the coupled heat transfer effect heat transfer on the aerogel material; (2) Accurately calculate the nanoscale solid thermal conductivity and the total effective thermal conductivity of nano-porous aerogel material with complex particle aggregation structures; (3) Study the characteristics of nanoscale radiative heat transfer as well as the impact of microstructure of the material on the nanoscale radiative heat transfer; (4) Optimize the structure design and preparation of aerogel material on the guide of the heat transfer mechanism of the material.

## Figures and Tables

**Table 1:** Characteristics of various heat-transfer models of aerogel.

Heat Transfer Mode	Characteristic
Gas heat transfer mode	The pore diameter is smaller than or close to the mean free path of gas molecules, thus greatly reducing the gas thermal conductivity.
Solid heat transfer mode	The characteristic scale of skeleton is close to the mean free path of solid phonons, thus greatly reducing the solid thermal conductivity.
Gas-solid coupled heat transfer mode	A large number of gas molecules are gathered in the contact surface of solid particles, forming gas-solid coupling heat transfer effect.
Radiative heat transfer mode	Aerogel has strong permeability to near-infrared radiation with wavelength of 3–8 μm at high temperature, which leads to poor shielding ability.

**Table 2:** The common methods for calculating the effective thermal conductivity.

The Common Methods	Disadvantages	Advantages
Decoupling method	The complex nano-porous structure of aerogel materials is ignored.	The method is simple in form and convenient in processing.
Equivalent circuit method	For different materials, the structural model needs to be rebuilt, which is a complicated method.	This method can reflect the influence of material structure and physical parameters on thermal conductivity.
Numerical simulation	The calculation method is complicated and only discrete numerical results can be obtained.	The method is accurate enough to take into account the influence of various factors on heat transfer.

**Table 3:** The common test methods.

The Test Methods		Disadvantages	Advantages
Heat conduction heating method	Steady state method	The test time is long and the test temperature is limited.	The test result is accurate.
	Unsteady state method	The test results are inaccurate and the test temperature is limited	The test time is short.
Infrared radiation heating method		This method can be used to test higher temperatures.	The involvement of infrared radiation leads to higher results.
Convection heating method		The cost of wind tunnel testing is higher.	The test result is accurate.

## Data Availability

We don’t have any publicly available data.

## References

[1] An L., Luigi M.D., Petit D., Hu Y., Chen Y., Armstrong J.N., Li Y.C., Ren S. (2022). Nanoengineering porous silica for thermal management. ACS Appl. Nano Mater..

[2] Li Z., Peng Y., Dong Y., Fan H., Chen P., Qiu L., Jiang Q. (2014). Effects of thermal efficiency in DCMD and the preparation of membranes with low thermal conductivity. Appl. Surf. Sci..

[3] Li C., Chen Z., Dong W., Lin L., Zhu X., Liu Q., Zhang Y., Zhai N., Zhou Z., Wang Y. (2021). A review of silicon-based aerogel thermal insulation materials: Performance optimization through composition and microstructure. J. Non-Cryst. Solids.

[4] He Y.-L., Xie T. (2015). Advances of thermal conductivity models of nanoscale silica aerogel insulation material. Appl. Therm. Eng..

[5] Hostler S.R., Abramson A.R., Gawryla M.D., Bandi S.A., Schiraldi D.A. (2009). Thermal conductivity of a clay-based aerogel. Int. J. Heat Mass Transf..

[6] Fesmire J.E. (2019). Aerogel-Based Insulation Materials for Cryogenic Applications. IOP Conf. Ser. Mater. Sci. Eng..

[7] Fu T., Tang J., Chen K., Zhang F. (2016). Determination of Scattering and Absorption Coefficients of Porous Silica Aerogel Composites. J. Heat Transf..

[8] Fu T., Tang J., Chen K., Zhang F. (2015). Visible, near-infrared and infrared optical properties of silica aerogels. Infrared Phys. Technol..

[9] Qu Z.G., Fu Y.D., Liu Y., Zhou L. (2018). Approach for predicting effective thermal conductivity of aerogel materials through a modified lattice Boltzmann method. Appl. Therm. Eng..

[10] Demilecamps A., Alves M., Rigacci A., Reichenauer G., Budtova T. (2016). Nanostructured interpenetrated organic-inorganic aerogels with thermal superinsulating properties. J. Non-Cryst. Solids.

[11] Hrubesh L.W., Pekala R.W. (2011). Thermal properties of organic and inorganic aerogels. J. Mater. Res..

[12] Merillas B., Vareda J.P., Martin-de Leon J., Rodriguez-Perez M.A., Duraes L. (2022). Thermal Conductivity of Nanoporous Materials: Where Is the Limit?. Polymers.

[13] Tang G.H., Bi C., Zhao Y., Tao W.Q. (2015). Thermal transport in nano-porous insulation of aerogel: Factors, models and outlook. Energy.

[14] Sen S., Singh A., Bera C., Roy S., Kailasam K. (2022). Recent developments in biomass derived cellulose aerogel materials for thermal insulation application: A review. Cellulose.

[15] Noroozi M., Panahi-Sarmad M., Abrisham M., Amirkiai A., Asghari N., Golbaten-Mofrad H., Karimpour-Motlagh N., Goodarzi V., Bahramian A.R., Zahiri B. (2019). Nanostructure of Aerogels and Their Applications in Thermal Energy Insulation. ACS Appl. Energy Mater..

[16] Saboktakin A., Saboktakin M.R. (2015). Improvements of reinforced silica aerogel nanocomposites thermal properties for architecture applications. Int. J. Biol. Macromol..

[17] Yang H., Ye F. (2022). Microtexture, microstructure evolution, and thermal insulation properties of Si(_3_)N(_4_)/silica aerogel composites at high temperatures. RSC Adv..

[18] Qian G., Wu B., Qin Z., Li X., Zheng Z., Xia R., Qian J. (2022). Enhanced Thermal Conductivity via In Situ Constructed CNT Aerogel Structure in Composites. Adv. Mater. Interfaces.

[19] Hayase G., Kugimiya K., Ogawa M., Kodera Y., Kanamori K., Nakanishi K. (2014). The thermal conductivity of polymethylsilsesquioxane aerogels and xerogels with varied pore sizes for practical application as thermal superinsulators. J. Mater. Chem. A.

[20] Calvert M., Baker J. (1998). Thermal Conductivity and Gaseous Microscale Transport. J. Thermophys. Heat Transf..

[21] Meng C., Yuan H. (2021). Examination on aerogel based on the heat transfer characteristics of nanotechnology. Ferroelectrics.

[22] Lallich S., Enguehard F., Baillis D. (2009). Experimental determination and modeling of the radiative properties of silica nanoporous matrices. J. Heat Transf..

[23] Liu D., Yan J.H., Wang F., Huang Q.X., Chi Y., Cen K.F. (2010). Inverse radiation analysis of simultaneous estimation of temperature field and radiative properties in a two-dimensional participating medium. Int. J. Heat Mass Transf..

[24] Torabi A., Abedian A., Farsi M.A. (2020). An approximate methodology to simulate combined conduction-radiation heat transfer for multi-layer insulator. Sci. Iran..

[25] Zhao J.-J., Duan Y.-Y., Wang X.-D., Wang B.-X. (2012). Effects of solid–gas coupling and pore and particle microstructures on the effective gaseous thermal conductivity in aerogels. J. Nanoparticle Res..

[26] Huang D., Shen Y., Yuan Q., Wang C., Shi L. (2019). Preparation and characterization of silica aerogel/polytetrafluoroethylene composites. Mater. Res. Express.

[27] Qu M.-L., Tian S.-Q., Fan L.-W., Yu Z.-T., Ge J. (2020). An experimental investigation and fractal modeling on the effective thermal conductivity of novel autoclaved aerated concrete (AAC)-based composites with silica aerogels (SA). Appl. Therm. Eng..

[28] Fricke J., Lu X., Wang P., Büttner D., Heinemann U. (1992). Optimization of monolithic silica aerogel insulants. Int. J. Heat Mass Transf..

[29] Smith B.R., Amon C.H. Effect of sub-continuum energy transport on effective thermal conductivity in nanoporous silica (Aerogel). Proceedings of the Asme International Mechanical Engineering Congress & Exposition.

[30] Yang M., Li X. (2022). Optimum convergence parameters of lattice Boltzmann method for predicting effective thermal conductivity. Comput. Methods Appl. Mech. Eng..

[31] Denpoh K. (1998). Modeling of rarefied gas heat conduction between wafer and susceptor. IEEE Trans. Semicond. Manuf..

[32] Kaganer M.G. (1969). Thermal Insulations in Cryogenic Engineering.

[33] Reichenauer G., Heinemann U., Ebert H.P. (2007). Relationship between pore size and the gas pressure dependence of the gaseous thermal conductivity. Colloids Surf. A Physicochem. Eng. Asp..

[34] Bi C., Tang G.H., Tao W.Q. (2012). Prediction of the gaseous thermal conductivity in aerogels with non-uniform pore-size distribution. J. Non-Cryst. Solids.

[35] Zeng S.Q., Hunt A.J., Cao W., Greif R. (1994). Pore size distribution and apparent gas thermal conductivity of silica aerogel. J. Heat Transf..

[36] Zeng S.Q., Hunt A.J., Greif R. (1995). Mean free path and apparent thermal conductivity of a gas in a porous medium. J. Heat Transf..

[37] Zeng S.Q., Hunt A.J., Greif R. (1995). Transport properties of gas in silica aerogel. J. Non-Cryst. Solids.

[38] Chen G. (1996). Nonlocal and nonequilibrium heat conduction in the vicinity of nanoparticles. J. Heat Transf..

[39] Wang B., Zhou L., Peng X. (2003). A fractal model for predicting the effective thermal conductivity of liquid with suspension of nanoparticles. Int. J. Heat Mass Transf..

[40] Zhao J.-J., Duan Y.-Y., Wang X.-D., Wang B.-X. (2012). A 3-D numerical heat transfer model for silica aerogels based on the porous secondary nanoparticle aggregate structure. J. Non-Cryst. Solids.

[41] Xie T., He Y.-L., Hu Z.-J. (2013). Theoretical study on thermal conductivities of silica aerogel composite insulating material. Int. J. Heat Mass Transf..

[42] Han Y.-F., Xia X.-L., Tan H.-P., Liu H.-D. (2013). Modeling of phonon heat transfer in spherical segment of silica aerogel grains. Phys. B Condens. Matter.

[43] Cahill D.G., Watson S.K., Pohl R.O. (1992). Lower limit to the thermal conductivity of disordered crystals. Phys. Rev. B Condens. Matter.

[44] Hopkins P.E., Kaehr B., Piekos E.S., Dunphy D., Jeffrey Brinker C. (2012). Minimum thermal conductivity considerations in aerogel thin films. J. Appl. Phys..

[45] Zhu C.-Y., Li Z.-Y. (2018). Modeling of the apparent solid thermal conductivity of aerogel. Int. J. Heat Mass Transf..

[46] Dames C., Chen G. (2004). Theoretical phonon thermal conductivity of Si/Ge superlattice nanowires. J. Appl. Phys..

[47] Bi C., Tang G.H. (2013). Effective thermal conductivity of the solid backbone of aerogel. Int. J. Heat Mass Transf..

[48] Obori M., Suh D., Yamasaki S., Kodama T., Saito T., Isogai A., Shiomi J. (2019). Parametric Model to Analyze the Components of the Thermal Conductivity of a Cellulose-Nanofibril Aerogel. Phys. Rev. Appl..

[49] Smith B.R., Beutler P.D., Amon C.H. Thermal transport network model for high-porosity materials: Application to nanoporous aerogels. Proceedings of the Asme Summer Heat Transfer Conference Collocated with the Asme Pacific Rim Technical Conference & Exhibition on Integration & Packaging of Mems.

[50] Pang H., Li Z. (2021). Experimental investigations on the thermal insulation performance of SiC opacifier doped silica aerogel at large temperature difference. Int. J. Therm. Sci..

[51] Swimm K., Reichenauer G., Vidi S., Ebert H.-P. (2017). Impact of thermal coupling effects on the effective thermal conductivity of aerogels. J. Sol-Gel Sci. Technol..

[52] Swimm K., Reichenauer G., Vidi S., Ebert H.P. (2009). Gas Pressure Dependence of the Heat Transport in Porous Solids with Pores Smaller than 10 μm. Int. J. Thermophys..

[53] Bi C., Tang G.H., Hu Z.J., Yang H.L., Li J.N. (2014). Coupling model for heat transfer between solid and gas phases in aerogel and experimental investigation. Int. J. Heat Mass Transf..

[54] Swimm K., Vidi S., Reichenauer G., Ebert H.P. (2017). Coupling of gaseous and solid thermal conduction in porous solids. J. Non-Cryst. Solids.

[55] Folgar C., Folz D., Suchicital C., Clark D. (2007). Microstructural evolution in silica aerogel. J. Non-Cryst. Solids.

[56] Liu H., Xia X., Xie X., Ai Q., Li D. (2017). Experiment and identification of thermal conductivity and extinction coefficient of silica aerogel composite. Int. J. Therm. Sci..

[57] He F., Qi Z., Zhen W., Wu J., Huang Y., Xiong X., Zhang R. (2019). Thermal Conductivity of Silica Aerogel Thermal Insulation Coatings. Int. J. Thermophys..

[58] Lou F., Dong S., Ma Y., Qi B., Zhu K. (2021). Numerical Study of the Influence of Coupling Interface Emissivity on Aerogel Metal Thermal Protection Performance. Gels.

[59] Lee O.J., Lee K.H., Yim T.J., Kim S.Y., Yoo K.P. (2002). Determination of mesopore size of aerogels from thermal conductivity measurements. J. Non-Cryst. Solids.

[60] Lu X., Arduini-Schuster M.C., Kuhn J., Nilsson O., Fricke J., Pekala R.W. (1992). Thermal conductivity of monolithic organic aerogels. Science.

[61] Lu X., Caps R., Fricke J., Alviso C.T., Pekala R.W. (1995). Correlation between structure and thermal conductivity aerogels. J. Non-Cryst. Solids.

[62] Lee S.-C., Cunnington G.R. (2000). Conduction and Radiation Heat Transfer in High-Porosity Fiber Thermal Insulation. J. Thermophys. Heat Transf..

[63] Zhang H., Wang X., Li Y. (2018). Measuring radiative properties of silica aerogel composite from FTIR transmittance test using KBr as diluents. Exp. Therm. Fluid Sci..

[64] Soorbaghi F.P., Kokabi M., Bahramian A.R. (2019). Predicting the effective thermal conductivity of silica/clay mineral nanocomposite aerogels. Int. J. Heat Mass Transf..

[65] Dai Y.-J., Tang Y.-Q., Fang W.-Z., Zhang H., Tao W.-Q. (2018). A theoretical model for the effective thermal conductivity of silica aerogel composites. Appl. Therm. Eng..

[66] Zeng S.Q., Hunt A., Greif R. (1995). Geometric structure and thermal conductivity of porous medium silica aerogel. J. Heat Transf..

[67] Wei G., Liu Y., Zhang X., Yu F., Du X. (2011). Thermal conductivities study on silica aerogel and its composite insulation materials. Int. J. Heat Mass Transf..

[68] He F., Wang Y., Zheng W., Wu J.-Y., Huang Y.-H. (2022). Effective thermal conductivity model of aerogel thermal insulation composite. Int. J. Therm. Sci..

[69] Dan D., Zhang H., Tao W.-Q. (2014). Effective structure of aerogels and decomposed contributions of its thermal conductivity. Appl. Therm. Eng..

[70] Liu H., Hu M., Jiao J., Li Z., Wu X. (2020). Effective thermal conductivity modeling of hollow nanosphere packing structures. Int. J. Heat Mass Transf..

[71] Liu H., Hu M., Jiao J., Li Z. (2020). Geometric optimization of aerogel composites for high temperature thermal insulation applications. J. Non-Cryst. Solids.

[72] Liu H., Li Z.Y., Zhao X.P., Tao W.Q. (2015). Study on Unit Cell Models and the Effective Thermal Conductivities of Silica Aerogel. J. Nanosci. Nanotechnol..

[73] Pia G., Sanna U. (2013). Intermingled fractal units model and electrical equivalence fractal approach for prediction of thermal conductivity of porous materials. Appl. Therm. Eng..

[74] Li Z.-Y., Liu H., Zhao X.-P., Tao W.-Q. (2015). A multi-level fractal model for the effective thermal conductivity of silica aerogel. J. Non-Cryst. Solids.

[75] Chen Y., Li D., Xie X.-Q., Gao Y., He Y.-L. (2020). Theoretical modeling and experimental validation for the effective thermal conductivity of moist silica aerogel. Int. J. Heat Mass Transf..

[76] Spagnol S., Lartigue B., Trombe A., Gibiat V. (2007). Thermal modeling of two-dimensional periodic fractal patterns, an application to nanoporous media. Europhys. Lett. (EPL).

[77] Bi C., Tang G.H., Hu Z.J. (2014). Heat conduction modeling in 3-D ordered structures for prediction of aerogel thermal conductivity. Int. J. Heat Mass Transf..

[78] Fang W.-Z., Zhang H., Chen L., Tao W.-Q. (2017). Numerical predictions of thermal conductivities for the silica aerogel and its composites. Appl. Therm. Eng..

[79] Han Y.-F., Liu H.-D., Chen X. (2018). Numerical simulation for thermal conductivity of nanograin within three dimensions. Therm. Sci..

[80] Kan A., Mao S., Wang N., Shi B. (2019). Simulation and Experimental Study on Thermal Conductivity of Nano-Granule Porous Material Based on Lattice-Boltzmann Method. J. Therm. Sci..

[81] Ross-Jones J., Gaedtke M., Sonnick S., Meier M., Rädle M., Nirschl H., Krause M.J. (2021). Pore-scale conjugate heat transfer simulations using lattice Boltzmann methods for industrial applications. Appl. Therm. Eng..

[82] Cheng L., Yue K., Wang J., Zhang X. (2017). A small-plane heat source method for measuring the thermal conductivities of anisotropic materials. Meas. Sci. Technol..

[83] Zhang H., Wu K., Tang G. (2021). Influence of Participating Radiation on Measuring Thermal Conductivity of Translucent Thermal Insulation Materials with Hot Strip Method. J. Therm. Sci..

[84] Zhang H., Li Y., Tao W. (2017). Effect of radiative heat transfer on determining thermal conductivity of semi-transparent materials using transient plane source method. Appl. Therm. Eng..

[85] Zhang H., Ma Y.X., Wang X., Tang G.H. (2021). Numerical study of the influence of thermal radiation on measuring semi-transparent thermal insulation material with hot wire method. Int. Commun. Heat Mass Transf..

[86] Zhang H., Fang W.-Z., Li Y.-M., Tao W.-Q. (2017). Experimental study of the thermal conductivity of polyurethane foams. Appl. Therm. Eng..

[87] Abdeali G., Bahramian A.R., Abdollahi M. (2020). Scale variation enhancement on heat transfer performance of cubic-like polymeric aerogel: With regard to structural parameters. Numer. Heat Transf. Part A Appl..

[88] Liu H., Xia X., Ai Q., Xie X., Sun C. (2017). Experimental investigations on temperature-dependent effective thermal conductivity of nanoporous silica aerogel composite. Exp. Therm. Fluid Sci..

[89] Yang J., Wu H., He S., Wang M. (2015). Prediction of thermal conductivity of fiber/aerogel composites for optimal thermal insulation. J. Porous Media.

[90] Bobda F., Claude Damfeu J., Ngono Mvondo R.R., Meukam P., Jannot Y. (2022). Thermal properties measurement of two tropical wood species as a function of their water content using the parallel hot wire method. Constr. Build. Mater..

[91] Peng F., Jiang Y., Feng J., Cai H., Feng J., Li L. (2021). Thermally insulating, fiber-reinforced alumina–silica aerogel composites with ultra-low shrinkage up to 1500 °C. Chem. Eng. J..

[92] Liu R.X., Cui T.Y., Zhou C.L., Wang C.H., Wang Y.Y., Sui X.Y. (2012). The Preparation and Assessment of the Rigid Aerogel Insulation Composites. Key Eng. Mater..

[93] Li Y., Zhang L., He R., Ma Y., Zhang K., Bai X., Xu B., Chen Y. (2019). Integrated thermal protection system based on C/SiC composite corrugated core sandwich plane structure. Aerosp. Sci. Technol..

[94] Lou F.F., Dong S.J., Zhao B.H., Qi B. (2022). Structuure design and performance test of a high temperature rise combustor based on combustion-gag wind tunnel device. Heat Transf. Res..

[95] Zhang R., Fan W. (2011). The design and performance of a high temperature rise combustor for wind tunnel. J. Therm. Sci..

